# A comparison of telehealth and in-person allied health practitioner service claims through the Medicare Benefits Schedule in Australia

**DOI:** 10.1177/20552076261434053

**Published:** 2026-03-18

**Authors:** Riley CC Brown, Megan H Ross, Centaine L Snoswell, Trevor G Russell

**Affiliations:** 1 RECOVER Injury Research Centre, 1974 The University of Queensland, Brisbane, Australia; 2 Centre for Research on Exercise, Physical Activity and Health (CRExPAH), School of Human Movement and Nutrition Sciences, 1974 The University of Queensland, Brisbane, Australia; 3 Centre for Online Health, 1974 The University of Queensland, Brisbane, Australia; 4 Centre for Health Services Research, 1974 The University of Queensland, Brisbane, Australia

**Keywords:** Videoconference, telephone, telerehabilitation, ecological, Medicare

## Abstract

**Background:**

In 2020, Australia introduced reimbursable telehealth services (videoconference and telephone) for allied health (AH) professionals in response to COVID-19 public health restrictions. This study aims to quantify and compare in-person, telephone and videoconference services for AH professionals before, during and after COVID-19 restrictions.

**Methods:**

This study uses publicly available Medicare Benefits Schedule (MBS) data from January 2017 to December 2024. Service counts for in-person, telephone, and videoconference consultations were extracted across key AH professions. An interrupted time-series analysis assessed all consultations before and after MBS AH telehealth item number introduction.

**Results:**

A significant increase in overall MBS AH services was observed in Q2 2020 (8% increase, *n* = 280,854 additional claims from Q1 2020), coinciding with telephone and videoconference item number introduction. There was no significant attrition for all MBS AH services through the extraction period (reduction of 0.05% per quarter). Telehealth modalities accounted for 24.9% of MBS AH services in Q2 2020, gradually declining to 9.6% by Q4 2024. Videoconference (70.2%) was more commonly claimed than telephone (29.8%) for AH telehealth services. Psychological interventions accounted for 95.5% (*n* = 6,382,680) of dedicated AH videoconference, and 74.1% (*n* = 2,277,371) of telephone services. Low uptake of telehealth services was noted for physiotherapy, exercise physiology and podiatry.

**Conclusions:**

Despite attrition through the extraction period, telephone and videoconference uptake contributed to a sustained increase in total AH service provision through the MBS. However, many AH services had low uptake. To ensure the long-term sustainability of telehealth, increased advocacy and promotion from the broader AH workforce is warranted.

## Introduction

Noncommunicable diseases (NCDs) and conditions, such as heart disease and type 2 diabetes, are an immense global health and financial burden. NCDs are attributable for 65% of deaths worldwide,^1^ with projections predicting a USD $47 trillion global economic burden between 2010 and 2030.^2^ It is anticipated that four out of five Australians will experience at least one long-term health condition during their lifetime, with 50% developing a NCD.^3^ Since 2004, the Australian Government's universal healthcare system, Medicare, has subsidised allied health (AH) services to support the management and prevention of NCDs. AH practitioners are university qualified and the majority are accredited by relevant national accreditation bodies (e.g. AH Practitioner Regulation Agency (AHPRA)) and have expertise in diagnosing, treating and preventing an array of NCDs.^4^ As such, AH services are a pivotal component of primary health care in Australia, with demonstrated links to improving patient outcomes and healthcare system cost effectiveness and efficiency.^5^ In 2024, 920,535 AH and medical practitioners were accredited through AHPRA,^6^ representing nearly a two-fold increase from 2014.

Largely due to federally funded initiatives and services provided through Medicare, Australia is in the top tier of countries worldwide for universal healthcare coverage.^7,8^ Despite this, access to AH services is often hampered by high societal demand and concentration of practitioners in and around large metropolitan centres.^4^ This disproportionately affects patients in rural and regional areas, and other disadvantaged communities.^9^ In March 2020, the Australian Government introduced telehealth item numbers (telephone and videoconference) to the Medicare Benefits Schedule (MBS) for AH services (made permanent in January 2022) in response to the COVID-19 pandemic.^10^ Telehealth services refer to the use of electronic communication and information technologies to deliver healthcare remotely.^11^ Previous research has demonstrated that telehealth services are clinically and cost-effective, across a range of settings and intervention types and is generally liked by patients.^12–15^

Reimbursement of telehealth services through the MBS coincided with an increase in total service counts for AH practitioners like dietitians,^16^ physiotherapists,^17^ exercise physiologists^18^ and psychologists.^19^ However, the impact of the introduction of telephone and videoconference MBS item numbers on the provision of all AH services is unknown. This information will help to inform our understanding of the uptake of telehealth services reimbursed through the MBS. Therefore, the aim of this study is to investigate trends across all AH in-person, telephone and videoconference services funded through the MBS over an eight-year timeframe (2017 to 2024).

## Methods

This study used publicly available MBS data to describe AH services reimbursed by Medicare between January 2017 and December 2024.^20^ This study received ethics exemption from The University of Queensland Human Research Ethics Committee (2020/HE001116).

Data were exported for all AH services with an in-person, telephone and videoconference item number from the Medicare Australia website.^20^ AH professions included in the extraction are presented in Table 1. All item numbers extracted for each profession are provided in Supplemental Material 1. Telephone and videoconference item numbers introduced in response to the COVID-19 pandemic (93000, 93048, 93537, 93592, 93013, 93061, 93538, 93593) do not differentiate between different AH professionals, but were included in the extracted list. Differentiation between AH professions for shared telephone and videoconference item numbers was based on overall percentage of Chronic Disease Management Plan claims in 2024 (Table 1). Annual MBS reports were searched to identify any in-person, telephone and videoconference item number codes that were active in the years 2017 to 2024.^21^ State/territory and local data were not sought for this analysis.

**Table 1.** Allied health service domains included in extraction from Medicare Australia website and percentage of total chronic disease management plan (CDMP) claims in 2024.

Allied health service domains	CDMP item number	% of total CDMP claims in 2024
1. Aboriginal or Torres Strait Islander health service	10950	0.03
2. Diabetes education	10951	1.1
3. Audiology	10952	0.04
4. Exercise physiology	10953	3.9
5. Dietetics	10954	4.9
6. Mental health services	10956	0.08
7. Occupational therapy	10958	0.9
8. Physiotherapy	10960	34.9
9. Podiatry	10962	42.5
10. Chiropractic	10964	6.6
11. Osteopathy	10966	3.2
12. Psychology	10968	0.4
13. Speech pathology	10970	1.5
14. Social work	N/A*	N/A*

*Social work not included in CDMP referral scheme and not allocated any portion of the shared allied health telephone or videoconference item numbers.

Data were exported from the Medicare Australia website to Microsoft Excel (2021, Microsoft Corp., Redmond, WA, USA) prior to analysis. Rates of service counts were reported as the quantity of services for each quarter of the year (Q1 2017 to Q4 2024). Data were analysed using descriptive statistics (including bar/line graphs) for quarterly services and presented according to delivery mode (in-person, videoconference and telephone). The average proportion (%) of in-person, telephone and videoconference service count was reported for each period. Analyses were completed using Microsoft Excel (Microsoft, Redmond, WA, USA).

An interrupted time-series regression (ITSR) analysis was conducted on the overall activity data in Stata v18.0 (SE) to examine whether the changes in volume and rate were statistically significant.^22^ ITSR has been used previously to quantify policy changes relating to COVID-19.^23,24^ An interruption was modelled at Q2 2020, when the new telephone and videoconference item numbers were introduced (introduction date: 30 March 2020). Trends were examined by comparing the changes in telephone and videoconference activity with previous time periods, and both coefficients and graphical representation of the analysis were derived. *p*-values of <.05 and 95% confidence intervals determined statistical significance.

## Results

**Figure 1.**
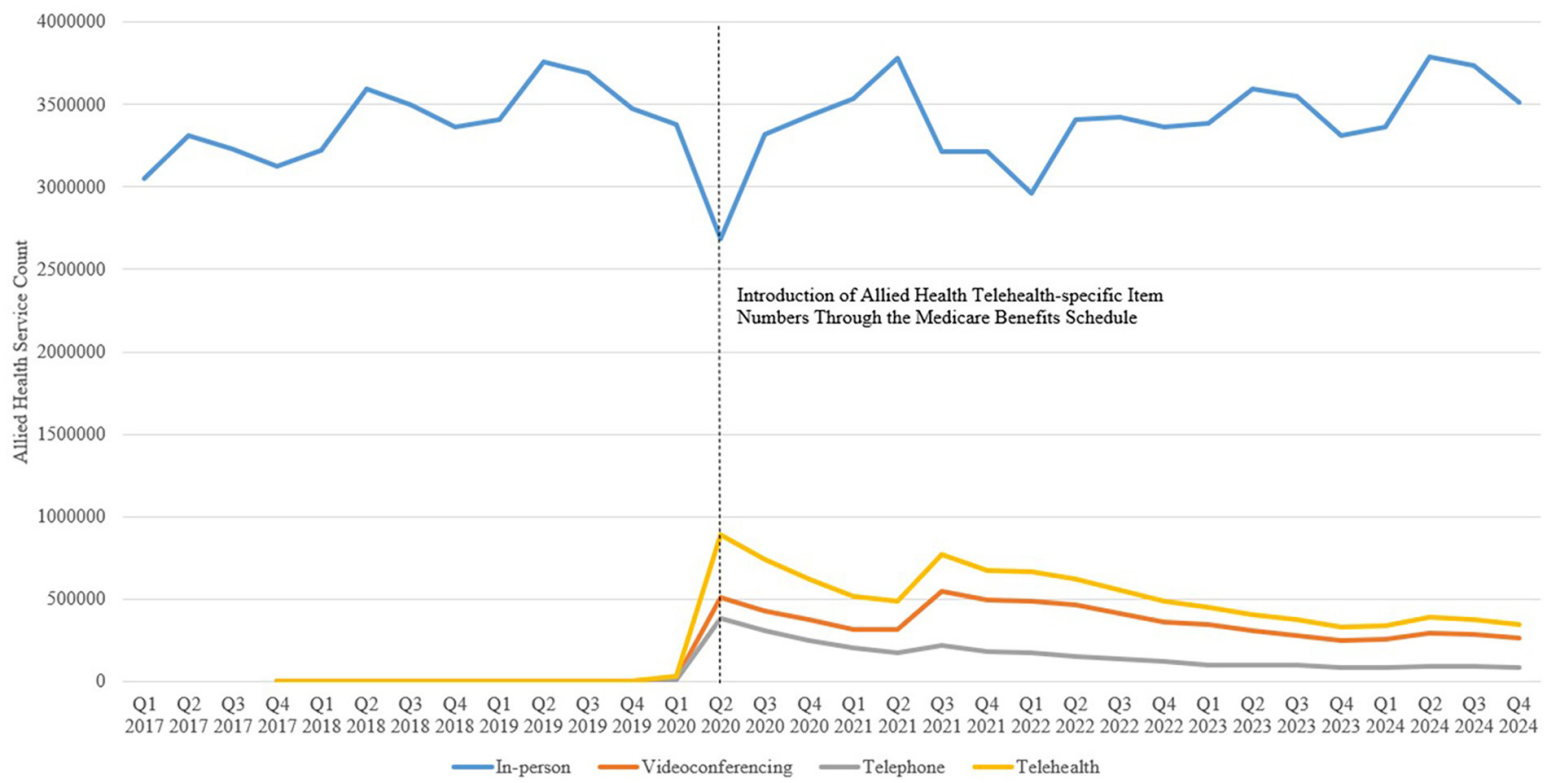
Quarterly claimed allied health services through the Medicare Benefits Schedule from Q1 2017 to Q4 2024, segmented into in-person, videoconferencing, telephone and telehealth (videoconferencing + telephone) modalities.

Quarterly data for all AH services through the MBS are presented in Table 2 and Figure 1. Service counts for each AH profession through the extraction period are presented in Supplemental Material 1. For profession-specific item numbers regardless of modality type, Psychology (40.0%, *n* = 47,576,288), Podiatry (23.7%, *n* = 28,199,494) and Physiotherapy (19.5%, *n* = 23,178,510) were the most commonly used AH services across the entire extraction period. Item number 10962 (In-person Individual Podiatry CDM Service) was the most claimed service, representing 14.1% (*n* = 27,827,268) of all AH services through the MBS. Item numbers 91167 (Videoconference Psychological Therapy Health Service – 50 min) and 91170 (Videoconference Focussed Psychological Health Strategies – 50 min) represented 51.4% of all telehealth-specific services.

**Table 2.** Allied health Medicare Benefits Schedule service counts (2017–2024).

Year	Quartile	In-person, *n*	Videoconference, *n*	Telephone, *n*	Total, *n*	Videoconference, %	Telephone, %	Telehealth, %
2017	Q1	3,050,087	0	0	3,050,087	0.0	0.0	0.0
	Q2	3,309,726	0	0	3,309,726	0.0	0.0	0.0
	Q3	3,232,211	0	0	3,232,211	0.0	0.0	0.0
	Q4	3,127,939	328	0	3,128,267	0.0	0.0	0.0
2018	Q1	3,223,681	862	0	3,224,543	0.0	0.0	0.0
	Q2	3,598,378	1224	0	3,599,602	0.0	0.0	0.0
	Q3	3,498,717	1259	0	3,499,976	0.0	0.0	0.0
	Q4	3,366,346	1907	0	3,368,253	0.1	0.0	0.1
2019	Q1	3,405,760	2529	0	3,408,289	0.1	0.0	0.1
	Q2	3,758,503	3239	0	3,761,742	0.1	0.0	0.1
	Q3	3,691,965	3631	0	3,695,596	0.1	0.0	0.1
	Q4	3,474,957	4191	0	3,479,148	0.1	0.0	0.1
2020	Q1	3,377,675	21,854	12,185	3,411,714	0.6	0.4	1.0
	Q2	2,683,651	506,701	384,488	3,574,840	14.2	10.8	24.9
	Q3	3,319,429	429,849	309,021	4,058,299	10.6	7.6	18.2
	Q4	3,430,921	374,227	245,827	4,050,975	9.2	6.1	15.3
2021	Q1	3,532,162	314,258	202,249	4,048,669	7.8	5.0	12.8
	Q2	3,782,550	314,084	176,578	4,273,212	7.4	4.1	11.5
	Q3	3,213,553	550,288	220,943	3,984,784	13.8	5.5	19.4
	Q4	3,217,923	492,737	184,336	3,894,996	12.7	4.7	17.4
2022	Q1	2,962,141	490,183	178,236	3,630,560	13.5	4.9	18.4
	Q2	3,409,477	463,403	155,490	4,028,370	11.5	3.9	15.4
	Q3	3,427,744	413,534	140,407	3,981,685	10.4	3.5	13.9
	Q4	3,367,829	361,623	124,182	3,853,634	9.4	3.2	12.6
2023	Q1	3,384,242	347,548	100,880	3,832,670	9.1	2.6	11.7
	Q2	3,593,429	308,227	100,815	4,002,471	7.7	2.5	10.2
	Q3	3,552,168	280,598	97,951	3,930,717	7.1	2.5	9.6
	Q4	3,315,542	245,957	86,249	3,647,748	6.7	2.4	9.1
2024	Q1	3,360,996	253,860	82,557	3,697,413	6.9	2.2	9.1
	Q2	3,791,945	296,505	91,461	4,179,911	7.1	2.2	9.3
	Q3	3,738,385	285,788	90,000	4,114,173	6.9	2.2	9.1
	Q4	3,514,486	260,498	85,962	3,860,946	6.7	2.2	9.0

*n*: number; Q1: Quartile one; Q2: Quartile two; Q3: Quartile three; Q4: Quartile four.

In-person services reduced by 20.5% in Q2 2020 (Figure 1). Telephone and videoconference modalities represented 24.9% of services in Q2 2020. After Q2 2020, in-person service count increased back to pre-restriction levels, averaging 3,439,720 services per quarter (87.1% of all AH MBS services) from Q3 2020 to Q4 2024. Telephone and videoconference services gradually declined after Q2 2020, averaging 614,146 services per quarter (15.4% of all AH MBS services) between Q3 2020 and Q4 2022, and 376,857 (9.6% of all AH MBS services) between Q1 2023 and Q4 2024.

**Figure 2.**
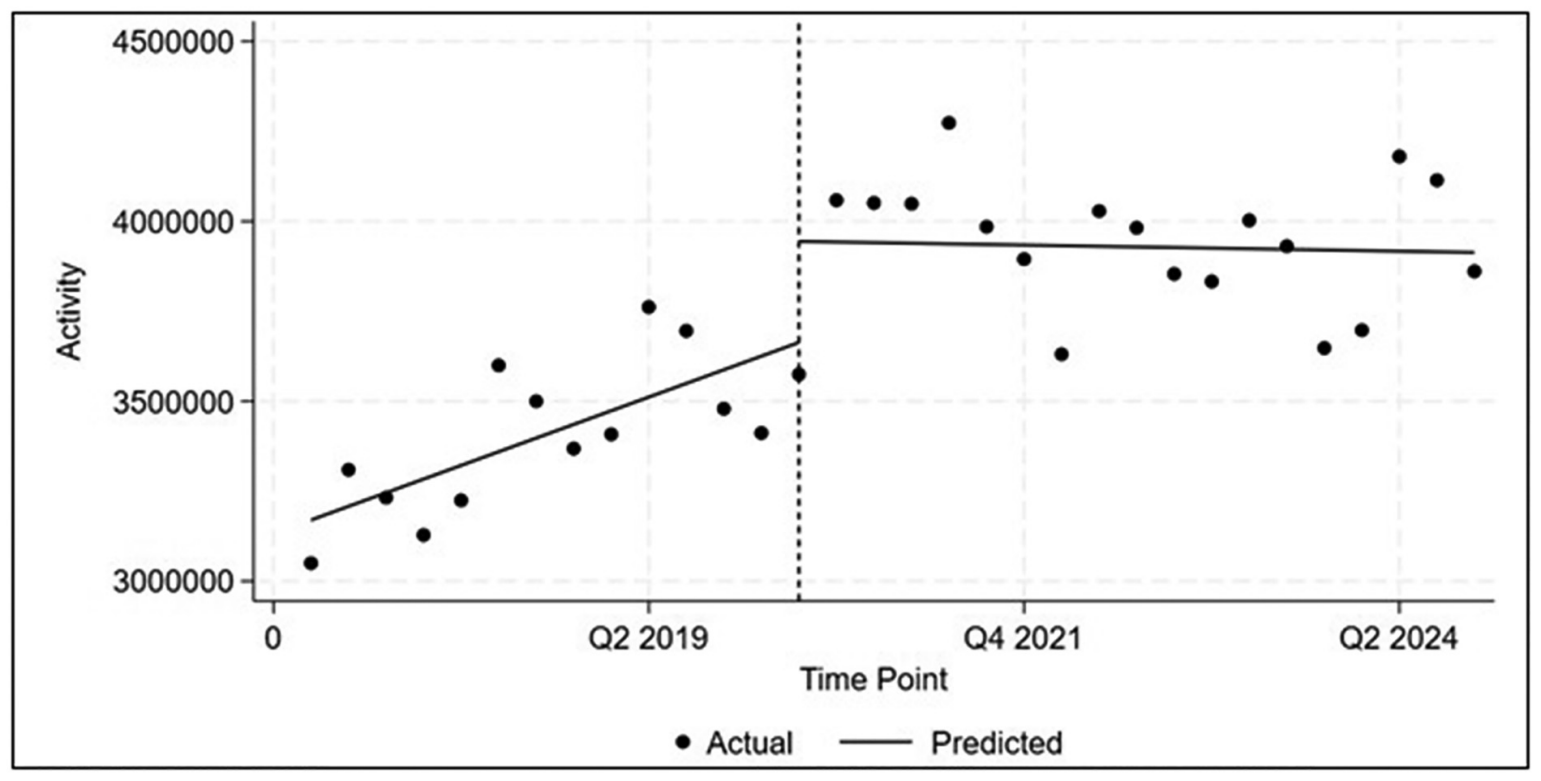
Changes in total activity after the introduction of new telehealth item numbers at Q2 2020: interrupted time-series regression analysis (*n* = 32 observations).

Comparison of AH service rates pre and post the introduction of telephone and videoconference MBS item numbers are displayed in Table 2. There was a significant increase in services after Q2 2020, largely attributed to additional claims through new telephone and videoconference codes (Figure 2). For AH professions with dedicated telephone and videoconference item numbers, telephone services were more common for Aboriginal or Torres Strait Islander Health Service (*n* = 493,587, 89.7% of telephone and videoconference services) and Audiology (*n* = 967, 64.4% of telehealth services). Videoconference delivery was more popular for Dietetics (*n* = 86,936, 91.6% of telehealth claims), Occupational Therapy (*n* = 31,760, 75.9%), Psychology (*n* = 5,990,847, 73.7%) and Social Work (*n* = 360,079, 73.5%). For shared telephone and videoconference item numbers that do not differentiate between AH professions (93000, 93048, 93537, 93592, 93013, 93061, 93538, 93593), telephone services (*n* = 289,778, 64.9%) were more commonly used. However, for all telehealth item numbers combined (profession-specific and non-differentiating), videoconference (*n* = 6,529,999, 70.2%) was more commonly claimed than telephone services (*n* = 2,771,039, 29.8%) (Supplemental Material). Psychological interventions for Psychologists (*n* = 5,990,847), Social Workers (*n* = 360,073) and Occupational Therapists (*n* = 31,760) accounted for 95.5% of profession-specific AH videoconference, and 74.1% (*n* = 2,277,371 overall) of telephone services (Table 2).

Prior to the introduction of the new item numbers, AH services were increasing steadily by 1.1% each quarter (∼37,950 claims) (Table 3, Figure 2). After the introduction of the new item numbers in Q2 2020, there was an immediate 8% service rate increase (280,854 additional services over activity in Q1 2020). From Q3 2020, activity reduced by 0.05% per quarter (1710 services) on average, however due to the quarterly variability this estimate is not statistically significant at α = 0.05.

**Table 3.** Changes in total activity after the introduction of new telehealth item numbers at Q2 2020: interrupted time-series regression analysis (*n* = 32 observations).

		Coefficient [95% CI]	*p*-value
Initial quarterly activity before Q2 2020	Level	3,169,925 [3,039,553 to 3,300,296]	<.000
	Slope (quarterly increase)	37,950 [13918 to 61983]	.002
Change in activity after Q2 2020	Level change (immediate increase)	280,854 [13,321 to 548,387]	.040
	Slope (ongoing quarterly decrease)	−1710 [−20842 to 17422]	.861
Total slope change pre- and post-Q2 2020		−39,661 [−73,185 to −6137]	.020

## Discussion

This ecological study aimed to describe the quantity of in-person, telephone and videoconference AH services reimbursed through the MBS from 2017 to 2024. The introduction of telephone and videoconference AH item numbers in Q2 2020 resulted in a significant and immediate increase in total service provision in Australia. This was despite a ∼20% reduction for in-person services. Despite slowly decreasing telehealth usage since Q3 2020, an overall increase in MBS AH service provision was sustained through the entire extraction period, compared to pre-pandemic levels. This observation may suggest that the introduction of telephone and videoconference modalities seems to have had a beneficial influence on AH service uptake through the MBS. Despite this total service increase, it was generally observed that uptake of telephone and videoconference modalities was minimal for most AH practitioners. Furthermore, psychological interventions delivered by Psychologists, Social Workers and Occupational Therapists represented the majority of telephone or videoconference AH services (95.5% of all telehealth services). However, most of these telephone and videoconference item numbers were established prior to pandemic-related physical distancing restrictions in Australia in Q2 2020. While they were seldom used nationally prior to pandemic restrictions, this indicates a sense of telehealth readiness for these intervention types.

Evidence suggests that synchronous psychological therapy delivered via telehealth appears to be as effective as in-person therapy for the treatment of depression and anxiety disorders.^25,26^ Attitudes towards telehealth delivery methods are generally positive for those delivering psychological services.^27,28^ A survey conducted during the COVID-19 pandemic in Australia and New Zealand identified that clinicians desired both in-person and telehealth options for the delivery of mental health services, tailored to patient circumstances and preferences.^28^ Clinicians reported low levels of opposition to telehealth adoption but identified perceived barriers to implementation. These included the presence of technological issues, safety concerns for patients with complex issues (e.g. trauma) and difficulties maintaining the therapeutic alliance when compared to in-person service.^28^ These observations are reflected in previous analyses of provider attitudes towards psychological telehealth services, where it was found to be an acceptable and important mode of treatment delivery with potential technological drawbacks.^27^ The higher volume of telehealth psychological services identified in this study, compared to other AH services (e.g. physiotherapy or exercise physiology services), may be partly explained by the perceived ease at which services can be implemented and scaled-up when compared to services that require a physical interaction, and the overall demand for psychological services increasing during COVID-19 restrictions. Negative clinician perceptions of safety, effectiveness and overall suitability of telehealth for individual practise have been identified as barriers to adoption for physiotherapists^29–32^ and exercise physiologists.^30,31,33,34^ However, evidence suggests that telehealth practise for exercise and physical rehabilitation is clinically and cost-effective and comparable to in-person practise.^12,14,15,35^ This potential disconnect between perceptions and outcomes may partially explain the minimal uptake of telephone and videoconference services for physiotherapists and exercise physiologists through the MBS. Additionally, experience and practise with telehealth has been proven to improve clinician confidence and willingness to use technology.^36^ The large number of psychological telephone and videoconference item numbers were available prior to the pandemic public health restrictions, this may help to explain the larger adoption rate.

Prior to Q2 2020, a steady increasing trend for total MBS AH activity was observed. The introduction of the MBS telephone and videoconference item numbers led to a short-term increase in service provision for a range of healthcare professionals.^16,18,19,24^ The current study suggests that this has been sustained for total AH service count through the MBS over a four-year period. However, attrition of telephone and videoconference AH services can be observed, with the provision rate of total telehealth services falling from 24.9% in Q2 2020 to 9.0% in Q4 2024. While it could be expected that the provision rate might have fallen after the lifting of physical distancing restrictions (Q2 2020) it is surprising how sharply telehealth activity has regressed. This is particularly surprising in professions where research suggests that telehealth services may be as clinically and cost-effective as in-person modalities (e.g. physiotherapy and exercise physiology) and where high patient satisfaction has been demonstrated.^12–15^ However, it must be noted that telehealth activity has not regressed to the negligible level seen pre-pandemic. It seems that telehealth has become embedded as a service delivery option for AH practitioners in Australia, with varying levels of uptake dependant on profession. Provider/patient preference, service suitability and item eligibility constraints likely influence uptake. Aboriginal and Torres Strait Islander Health Services is the only AH profession with marked and sustained telehealth activity that has remained higher than in-person service usage throughout the extraction period. This was largely driven by telephone-specific item numbers for chronic disease follow-up appointments (item number 93203). Further investigation into trends and potential mechanisms for uptake should be conducted.

Given the immediate and sustained increase in overall AH services, it is possible that access to services through telehealth improved overall awareness for the benefits of AH interventions for patients in Australia, leading to continuation of service once pandemic restrictions had lifted. This may be underscored by the observation that total AH service provision increased overall post-restrictions, despite many AH professions having minimal telehealth uptake. Potential reasons for this may include an increased desire for in-person services post-restrictions and increased demand for AH services overall.^17^ These findings may provide further evidence for the integration of hybrid health care delivery models for AH practitioners, including a combination of both in-person and telehealth services. Post Q2 2020 trends indicate a slight decrease in the growth of total AH activity through the MBS. While not statistically significant, this observation is stark compared to the pre-restriction period. The difference in growth questions whether telehealth modalities are leading to sustained and long-term growth for AH services through the MBS and should be investigated further. Given the low uptake in relevant professions, it should be recognised that all AH practitioners have a substantial role to play in the adoption and promotion of telehealth services so they remain a viable and attractive service reimbursement option.

For telephone and videoconference services to be sustainable for AH practitioners over the long-term, certain universal criteria must be met for administration, delivery and structure. These criteria include: (i) developing a skilled workforce competent in the delivery of telehealth techniques and services relevant to the profession, (ii) empowering consumers to advocate for telehealth modalities to reflect personal preferences and individualisation, (iii) continuation and expansion of funding models to include various forms of telehealth, and (iv) integrate telehealth into routine care and clinical workflows whilst considering factors to enhance staff willingness (e.g. sense of ownership).^37^ Alignment of services to these requirements may help to sustain telehealth modalities as long-term and viable forms of AH service delivery.

This study has several important limitations to consider. Data were extracted at a national level and analysis by local area or specific population groups was outside the scope of this assessment. This masks substantial jurisdictional differences at the state/territory and local level, including varying lockdown durations, workforce disruptions and reopening timelines. In turn, the interpretability of the generated data is diminished for separate jurisdictions and should be interpreted with caution. Additionally, local AH practitioners are required for in-person service provision and are more likely to reside in metropolitan areas. Telehealth services are likely to increase accessibility for rural and regional areas, but this could not be determined from the publicly available data used for this analysis. In turn, this limits inference regarding access and equity for rural and remote communities and socioeconomic status, however future research could explore this. The data we present are only for AH services provided through the MBS with both an in-person and telehealth (telephone or videoconference) equivalent item number. Most AH services are provided privately, through private health insurers or other government agencies and hospitals. Therefore, this study is not reflective of all AH services provided in Australia from 2017 to 2024. Additionally, the analyses conducted in this study do not account for natural population growth. This study only interprets service count and does not give an indication of AH service quality, clinical effectiveness, or overall cost effectiveness for Medicare or society.

## Conclusion

This study provides an overview of in-person, telephone and videoconference AH services conducted through the MBS from 2017 to 2024. The introduction of AH telephone and videoconference item numbers saw an immediate and significant increase in overall AH service provision. While overall service provision was sustained after the introduction of the new item numbers, there was marked attrition of telephone and videoconference services through the extraction period. The vast majority of AH telehealth services were psychological interventions provided by Psychologists, Social Workers and Occupational Therapists, with minimal uptake from other professional groups (e.g. Physiotherapy and Exercise Physiology). Future work is needed to investigate the reasons for low uptake given the potential of telehealth to improve accessibility and availability of AH services.

## Supplemental Material

sj-pdf-1-dhj-10.1177_20552076261434053 - Supplemental material for A comparison of telehealth and in-person allied health practitioner service claims through the Medicare Benefits Schedule in Australia Supplemental material, sj-pdf-1-dhj-10.1177_20552076261434053 for A comparison of telehealth and in-person allied health practitioner service claims through the Medicare Benefits Schedule in Australia by Riley CC Brown, Megan H Ross, Centaine L Snoswell and Trevor G Russell in DIGITAL HEALTH
