# Supplementary material for: A comparison of telehealth and in-person allied health practitioner service claims through the Medicare Benefits Schedule in Australia
Source: Digit Health. 2026 Mar 18;12:20552076261434053. doi: 10.1177/20552076261434053 (PMC13009995; doi:10.1177/20552076261434053)
Supplement: sj-pdf-1-dhj-10.1177_20552076261434053 - Supplemental material for A comparison of telehealth and in-person allied health practitioner service claims through the Medicare Benefits Schedule in Australia [file sj-pdf-1-dhj-10.1177_20552076261434053.pdf]

A Comparison of Telehealth and In-Person Allied Health Practitioner Service Claims through the Medicare Benefits Schedule in Australia

Online Supplementary Material

Contents

Supplementary Material 1: Allied Health Item Numbers through the Medicare Benefits Schedule Extracted from the Medicare Australia Website 2

Supplementary Table S1: Aboriginal or Torres Strait Islander Health Service 2

Supplementary Table S2: Diabetes education 2

Supplementary Table S3: Audiology 2

Supplementary Table S4: Exercise Physiology 3

Supplementary Table S5: Dietetics 3

Supplementary Table S6: Mental health 4

Supplementary Table S7: Occupational therapy 4

Supplementary Table S8: Physiotherapy 6

Supplementary Table S9: Podiatry 7

Supplementary Table S10: Chiropractic 7

Supplementary Table S11: Osteopathy 7

Supplementary Table S12: Psychology 8

Supplementary Table S13: Speech pathology 12

Supplementary Table S14: Other items extracted 12

Supplementary Table S15: Combined allied health telehealth item numbers 15

Supplementary Material 2 Quarterly Claimed Services through the Medicare Benefits Schedule from Q1 2017 to Q4 2024, Segmented into In-person, Videoconferencing, Telephone and Telehealth (Videoconferencing + Telephone) Modalities for All Allied Health Practitioners 16

Supplementary Figure S1 Aboriginal or Torres Strait Islander Health Service 16

Supplementary Figure S2 Diabetes Education 17

Supplementary Figure S3 Audiology 18

Supplementary Figure S4 Exercise Physiology 19

Supplementary Figure S5 Dietetics 20

Supplementary Figure S6 Mental Health Services 21

Supplementary Figure S7 Occupational Therapy 22

Supplementary Figure S8 Physiotherapy 23

Supplementary Figure S9 Podiatry 24

Supplementary Figure S10 Chiropractic 25

Supplementary Figure S11 Osteopathy 26

Supplementary Figure S12 Psychology 27

Supplementary Figure S13 Speech Pathology 28

Supplementary Figure S14 Social Work 29

Supplementary Material 1: Allied Health Item Numbers through the Medicare Benefits Schedule Extracted from the Medicare Australia Website

Supplementary Table S1: Aboriginal or Torres Strait Islander Health Service

Mode	Medicare Item Number	Title
In-person	10950	Aboriginal or Torres Strait Islander Health Service CDM
In-person	81300	Aboriginal or Torres Strait Islander Health Service (≥20min)
In-person	10984	Aboriginal or Torres Strait Islander Health Service telehealth assistance from RACF
Videoconference	93200	Aboriginal or Torres Strait Islander Health Service follow up (health check)
Videoconference	93201	Aboriginal or Torres Strait Islander Health Service follow up (chronic disease)
Phone	93202	Aboriginal or Torres Strait Islander Health Service follow up (health check)
Phone	93203	Aboriginal or Torres Strait Islander Health Service follow up (chronic disease)
In-person	93501	Aboriginal or Torres Strait Islander Health Service RACF assessment
In-person	93524	Aboriginal or Torres Strait Islander Health Service RACF subsequent service
In-person	93546	Aboriginal or Torres Strait Islander Health Service RACF for someone of Aboriginal or Torres Strait Islander descent assessment
In-person	93579	Aboriginal or Torres Strait Islander Health Service RACF for someone of Aboriginal or Torres Strait Islander descent subsequent service

Supplementary Table S2: Diabetes education

Mode	Medicare Item Number	Title
In-person	10951	Diabetes educator CDM
In-person	81305	Diabetes educator Aboriginal or Torres Strait Islander
In-person	93502	Diabetes educator RACF assessment
In-person	93525	Diabetes educator RACF subsequent service
In-person	93547	Diabetes educator Aboriginal or Torres Strait Islander RACF assessment
In-person	93580	Diabetes educator Aboriginal or Torres Strait Islander RACF subsequent service

Supplementary Table S3: Audiology

Mode	Medicare Item Number	Title
In-person	10952	Audiology CDM

In-person	81310	Audiology Aboriginal or Torres Strait Islander
In-person	82301	Audiology programming an auditory implant or the sound processor of an auditory implant (unilateral)
Videoconference	82302	Audiology programming an auditory implant or the sound processor of an auditory implant (unilateral)
Phone	82304	Audiology programming an auditory implant or the sound processor of an auditory implant (unilateral)
In-person	93503	Audiology RACF assessment
In-person	93526	Audiology RACF service
In-person	93548	Audiology Aboriginal or Torres Strait Islander RACF assessment
In-person	93581	Audiology Aboriginal or Torres Strait Islander RACF service

Supplementary Table S4: Exercise Physiology

Mode	Medicare Item Number	Title
In-person	10953	Exercise Physiology CDM
In-person	81315	Exercise Physiology Aboriginal or Torres Strait Islander
In-person	93504	Exercise Physiology RACF assessment
In-person	93518	Exercise Physiology RACF
In-person	93527	Exercise Physiology RACF subsequent service
In-person	93549	Exercise Physiology Aboriginal or Torres Strait Islander RACF assessment
In-person	93571	Exercise Physiology Aboriginal or Torres Strait Islander RACF
In-person	93582	Exercise Physiology Aboriginal or Torres Strait Islander RACF subsequent service

Supplementary Table S5: Dietetics

Mode	Medicare Item Number	Title
In-person	10954	Dietetics CDM
In-person	81120	Dietetics type 2 diabetes group assessment
In-person	81125	Dietetics type 2 diabetes group service
In-person	81320	Dietetics Aboriginal or Torres Strait Islander service
In-person	82350	Dietetics eating disorder management plan service
Videoconference	82351	Dietetics eating disorder management plan service
Videoconference	93074	Dietetics eating disorder management plan service

Phone	93108	Dietetics eating disorder management plan service
Videoconference	93284	Dietetics type 2 diabetes group assessment
Videoconference	93285	Dietetics type 2 diabetes group service
Phone	93286	Dietetics type 2 diabetes group assessment
In-person	93505	Dietetics RACF assessment
In-person	93528	Dietetics RACF service
In-person	93550	Dietetics Aboriginal or Torres Strait Islander RACF assessment
In-person	93583	Dietetics Aboriginal or Torres Strait Islander RACF service

Supplementary Table S6: Mental health

**Mode**	**Medicare Item Number**	**Title**
In-person	10956	Mental health CDM
In-person	81325	Aboriginal or Torres Strait Islander mental health service
In-person	93506	Mental health RACF assessment
In-person	93529	Mental health RACF service
In-person	93551	Mental health Aboriginal or Torres Strait Islander RACF assessment
In-person	93584	Mental health Aboriginal or Torres Strait Islander RACF service

Supplementary Table S7: Occupational therapy

**Mode**	**Medicare Item Number**	**Title**
In-person	10958	OT CDM
In-person	80125	OT focussed psychological strategies health service (20-50min) in consultation room + report
In-person	80129	OT focussed psychological strategies (20-50min) in consultation room to a person other than the patient
In-person	80130	OT focussed psychological strategies (20-50min) outside consultation room + report
In-person	80131	OT focussed psychological strategies (20-50min) outside consultation room to a person other than the patient
In-person	80135	OT focussed psychological strategies (>50min) in consultation room + report
In-person	80137	OT focussed psychological strategies (>50min) in consultation room to a person other than the patient
In-person	80140	OT focussed psychological strategies (>50min) outside consultation room + report

In-person	80141	OT focussed psychological strategies (>50min) outside consultation room to a person other than the patient
In-person	80145	OT focussed psychological strategies (≥60min) group (4-10 patients)
Videoconference	80146	OT focussed psychological strategies (≥60min) group (4-10 patients)
In-person	80147	OT focussed psychological strategies (≥90min) group
Videoconference	80148	OT focussed psychological strategies (≥90min) group
In-person	80152	OT focussed psychological strategies (≥120min) group
Videoconference	80153	OT focussed psychological strategies (≥120min) group
In-person	82010	OT complex neurodevelopmental disorder or eligible disability (≥50min)
In-person	82025	OT complex neurodevelopmental disorder or eligible disability (≥30min)
In-person	81330	OT health service for Aboriginal or Torres Strait Islander (≥20min)
In-person	82368	OT eating disorder psychological treatment (20-50min) in consultation room
In-person	82370	OT eating disorder psychological treatment (20-50min) outside consultation room
In-person	82371	OT eating disorder psychological treatment (≥50min) in consultation room
In-person	82373	OT eating disorder psychological treatment (≥50min) outside consultation room
In-person	82374	OT eating disorder psychological treatment (≥60min) group (6-10 patients)
Videoconference	82375	OT eating disorder psychological treatment (≥60min) group (6-10 patients)
Videoconference	91172	OT focussed psychological strategies (20-50min) + report
Videoconference	91173	OT focussed psychological strategies (≥50min) + report
Videoconference	91194	OT focussed psychological strategies (20-50min)
Videoconference	91195	OT focussed psychological strategies (≥50min)
Phone	91185	OT focussed psychological strategies (20-50min) + report
Phone	91186	OT focussed psychological strategies (≥50min) + report
Phone	91202	OT focussed psychological strategies (20-50min)
Phone	91203	OT focussed psychological strategies (≥50min)
Videoconference	93092	OT eating disorder psychological treatment (20-50min)
Videoconference	93095	OT eating disorder psychological treatment (≥50min)
Phone	93126	OT eating disorder psychological treatment (20-50min)
Phone	93129	OT eating disorder psychological treatment (≥50min)
Videoconference	80126	OT focussed psychological strategies (20-50min)
Videoconference	80136	OT focussed psychological strategies (≥50min)
Videoconference	82369	OT eating disorder psychological (20-50min)

Videoconference	82372	OT eating disorder psychological (≥50min)
In-person	91125	OT focussed psychological strategies in consultation room (bushfire) (20-50min)
Videoconference	91126	OT focussed psychological strategies (bushfire) (20-50min)
In-person	91130	OT focussed psychological strategies outside consultation room (bushfire) (20-50min)
In-person	91135	OT focussed psychological strategies in consultation room (bushfire) (≥50min)
Videoconference	91136	OT focussed psychological strategies in consultation room (bushfire) (≥50min)
In-person	91140	OT focussed psychological strategies outside consultation room (bushfire) (≥50min)
In-person	93322	OT focussed psychological strategies RACF (20-50min)
In-person	93323	OT focussed psychological strategies RACF (≥50min)
In-person	93356	OT focussed psychological strategies additional mental health (20-50min)
Videoconference	93357	OT focussed psychological strategies additional mental health (20-50min)
Phone	93358	OT focussed psychological strategies additional mental health (20-50min)
In-person	93359	OT focussed psychological strategies additional mental health (≥50min)
Videoconference	93360	OT focussed psychological strategies additional mental health (≥50min)
Phone	93361	OT focussed psychological strategies additional mental health (≥50min)
In-person	93383	OT focussed psychological strategies RACF mental health (20-50min)
In-person	93384	OT focussed psychological strategies RACF mental health (≥50min)
In-person	93507	OT RACF assessment
In-person	93519	OT RACF additional service
In-person	93530	OT RACF service
In-person	93552	OT Aboriginal or Torres Strait Islander RACF service
In-person	93572	OT Aboriginal or Torres Strait Islander additional RACF service
In-person	93585	OT Aboriginal or Torres Strait Islander subsequent RACF service

Supplementary Table S8: Physiotherapy

**Mode**	**Medicare Item Number**	**Title**
In-person	10960	Physiotherapy CDM
In-person	81335	Physiotherapy Aboriginal or Torres Strait Islander service
In-person	93508	Physiotherapy RACF assessment
In-person	93520	Physiotherapy RACF service

In-person	93531	Physiotherapy RACF subsequent service
In-person	93553	Physiotherapy Aboriginal or Torres Strait Islander RACF assessment
In-person	93573	Physiotherapy Aboriginal or Torres Strait Islander RACF service
In-person	93586	Physiotherapy Aboriginal or Torres Strait Islander RACF subsequent service

Supplementary Table S9: Podiatry

Mode	Medicare Item Number	Title
In-person	10962	Podiatry CDM
In-person	81340	Podiatry Aboriginal or Torres Strait Islander service
In-person	93509	Podiatry RACF assessment
In-person	93532	Podiatry RACF service
In-person	93554	Podiatry Aboriginal or Torres Strait Islander RACF assessment
In-person	93587	Podiatry Aboriginal or Torres Strait Islander RACF service

Supplementary Table S10: Chiropractic

Mode	Medicare Item Number	Title
In-person	10964	Chiropractic CDM
In-person	81345	Chiropractic Aboriginal or Torres Strait Islander service
In-person	93510	Chiropractic RACF assessment
In-person	93533	Chiropractic RACF service
In-person	93555	Chiropractic Aboriginal or Torres Strait Islander RACF assessment
In-person	93588	Chiropractic Aboriginal or Torres Strait Islander RACF service

Supplementary Table S11: Osteopathy

Mode	Medicare Item Number	Title
In-person	10966	Osteopathy CDM
In-person	81350	Osteopathy Aboriginal or Torres Strait Islander service
In-person	93511	Osteopathy RACF assessment
In-person	93534	Osteopathy RACF service

In-person	93556	Osteopathy Aboriginal or Torres Strait Islander RACF assessment
In-person	93589	Osteopathy Aboriginal or Torres Strait Islander RACF service

Supplementary Table S12: Psychology

Mode	Medicare Item Number	Title
In-person	10968	Psychology CDM
In-person	80000	Psychological therapy health service (30-50min) in consultation room
Videoconference	80001	Psychological therapy health service (30-50min)
In-person	80002	Psychological therapy health service (30-50min) in consultation room to a person other than the patient
In-person	80005	Psychological therapy health service (30-50min) outside consultation room
In-person	80006	Psychological therapy health service (30-50min) outside consultation room to a person other than the patient
In-person	80010	Psychological therapy health service (50min) in consultation room
Videoconference	80011	Psychological therapy health service (50min)
In-person	80012	Psychological therapy health service (50min) in consultation room to a person other than the patient
In-person	80015	Psychological therapy health service (50min) outside consultation room + report
In-person	80016	Psychological therapy health service (50min) outside consultation room to a person other than the patient
In-person	80020	Psychological therapy health service (≥60min) group (4-10 patients)
Videoconference	80021	Psychological therapy health service (≥60min) group (4-10 patients)
In-person	80022	Psychological therapy health service (≥90min) group (4-10 patients)
Videoconference	80023	Psychological therapy health service (≥90min) group (4-10 patients)
In-person	80024	Psychological therapy health service (≥120min) group (4-10 patients)
Videoconference	80025	Psychological therapy health service (≥120min) group (4-10 patients)
In-person	80100	Focussed psychological strategies health service (20-50min) in consultation room
Videoconference	80101	Focussed psychological strategies health service (20-50min)
In-person	80102	Focussed psychological strategies health service (20-50min) in consultation room to a person other than the patient
In-person	80105	Focussed psychological strategies health service (20-50min) outside consultation room
In-person	80106	Focussed psychological strategies health service (20-50min) outside consultation room to a person other than the patient
In-person	80110	Focussed psychological strategies health service (≥50min) in consultation room

Videoconference	80111	Focussed psychological strategies health service (≥50min)
In-person	80112	Focussed psychological strategies health service (≥50min) in consultation room to a person other than the patient
In-person	80115	Focussed psychological strategies health service (≥50min) outside consultation room
In-person	80116	Focussed psychological strategies health service (≥50min) outside consultation room to a person other than the patient
In-person	80120	Focussed psychological strategies health service (≥60min) group (4-10 patients)
Videoconference	80121	Focussed psychological strategies health service (≥60min) group (4-10 patients)
In-person	80122	Focussed psychological strategies health service (≥90min) group (4-10 patients)
Videoconference	80123	Focussed psychological strategies health service (≥90min) group (4-10 patients)
In-person	80127	Focussed psychological strategies health service (≥120min) group (4-10 patients)
Videoconference	80128	Focussed psychological strategies health service (≥120min) group (4-10 patients)
In-person	81000	Pregnancy support counselling (≥30min)
In-person	82000	Psychology health service for under 25 yrs old (≥50min)
In-person	82015	Psychology health service for complex neurodevelopmental disorder or eligible disability (≥30min)
In-person	81355	Psychology health service Aboriginal or Torres Strait Islander (20 min) chronic condition
In-person	82352	Eating disorder psychological treatment service (30-50min) in consultation room
Videoconference	82353	Eating disorder psychological treatment service (30-50min)
In-person	82354	Eating disorder psychological treatment service (30-50min) outside consultation room
In-person	82355	Eating disorder psychological treatment service (≥50min) in consultation room
Videoconference	82356	Eating disorder psychological treatment service (≥50min)
In-person	82357	Eating disorder psychological treatment service (≥50min) outside consultation room
In-person	82358	Eating disorder psychological treatment service (≥60min) group (6-10 patients) (clin psyc)
Videoconference	82359	Eating disorder psychological treatment service (≥60min) group (6-10 patients) (clin psyc)
In-person	82360	Eating disorder psychological treatment service (20-50min) in consultation room
Videoconference	82361	Eating disorder psychological treatment service (20-50min)
In-person	82362	Eating disorder psychological treatment service (20-50min) outside consultation room (clin psyc)
In-person	82363	Eating disorder psychological treatment service (≥50min) in consultation room (clin psyc)
Videoconference	82364	Eating disorder psychological treatment service (≥50min)
In-person	82365	Eating disorder psychological treatment service (≥50min) outside consultation room (clin psyc)
In-person	82366	Eating disorder psychological treatment service (≥60min) group (6-10 patients)
Videoconference	82367	Eating disorder psychological treatment service (≥60min) group (6-10 patients)

In-person	91000	Psychological therapy health service in consultation room (30-50min) – bushfire
Videoconference	91001	Psychological therapy health service (30-50min) – bushfire
In-person	91005	Psychological therapy health service outside consultation room (30-50min) – bushfire
In-person	91010	Psychological therapy health service in consultation room (≥50min) – bushfire
Videoconference	91011	Psychological therapy health service in consultation room (≥50min) – bushfire
In-person	91015	Psychological therapy health service outside consultation room (≥50min) – bushfire
In-person	91100	Focussed psychological strategies in consultation room (20-50min) – bushfire
Videoconference	91101	Focussed psychological strategies in consultation room (20-50min) – bushfire
In-person	91105	Focussed psychological strategies outside consultation room (20-50min) – bushfire
In-person	91110	Focussed psychological strategies in consultation room (50min) – bushfire
Videoconference	91111	Focussed psychological strategies in consultation room (50min) – bushfire
In-person	91115	Focussed psychological strategies outside consultation room (50min) – bushfire
Videoconference	91166	Psychological therapy health service (30-50min)
Videoconference	91167	Psychological therapy health service (≥50min)
Videoconference	91168	Psychological therapy health service to someone other than the patient (30-50min)
Videoconference	91171	Psychological therapy health service to someone other than the patient (≥50min)
Videoconference	91169	Focussed psychological health strategies (20-50min)
Videoconference	91170	Focussed psychological health strategies (≥50min)
Videoconference	91174	Focussed psychological health strategies to someone other than the patient (30-50min)
Videoconference	91177	Focussed psychological health strategies to someone other than the patient (≥50min)
Phone	91181	Psychological therapy health service (30-50min) + report
Phone	91182	Psychological therapy health service (≥50min) + report
Phone	91198	Psychological therapy health service (30-50min) to someone other than the patient
Phone	91199	Psychological therapy health service (≥50min) to someone other than the patient
Phone	91183	Focussed psychological health strategies (20-50min) + report
Phone	91184	Focussed psychological health strategies (≥50min) + report
Phone	91200	Focussed psychological health strategies (20-50min) to someone other than the patient
Phone	91201	Focussed psychological health strategies (≥50min) to someone other than the patient
Videoconference	93026	Pregnancy support counselling (30min)
Phone	93029	Pregnancy support counselling (30min)
Videoconference	93032	Psychology health service for under 25 yrs old (≥50min)

Videoconference	93035	Psychology health service for complex neurodevelopmental disorder or eligible disability (≥30min)
Phone	93040	Psychology health service for under 25 yrs old (≥50min)
Phone	93043	Psychology health service for complex neurodevelopmental disorder or eligible disability (≥30min)
Videoconference	93076	Eating disorder psychological treatment service (30-50min) (clin psyc)
Videoconference	93079	Eating disorder psychological treatment service (≥50min) (clin psyc)
Videoconference	93084	Eating disorder psychological treatment service (20-50min)
Videoconference	93087	Eating disorder psychological treatment service (≥50min)
Phone	93110	Eating disorder psychological treatment service (30-50min) (clin psyc)
Phone	93113	Eating disorder psychological treatment service (≥50min) (clin psyc)
Phone	93118	Eating disorder psychological treatment service (20-50min)
Phone	93121	Eating disorder psychological treatment service (≥50min)
In-person	93312	Psychological therapy health service RACF (30-50min)
In-person	93313	Psychological therapy health service RACF (50min)
In-person	93316	Focussed psychological strategies RACF (20-50min)
In-person	93319	Focussed psychological strategies RACF (50min)
In-person	93330	Psychological therapy health service additional mental health service (30-50min)
Videoconference	93331	Psychological therapy health service additional mental health service (30-50min)
Phone	93332	Psychological therapy health service additional mental health service (30-50min)
In-person	93333	Psychological therapy health service additional mental health service (50min)
Videoconference	93334	Psychological therapy health service additional mental health service (50min)
Phone	93335	Psychological therapy health service additional mental health service (50min)
In-person	93350	Focussed psychological strategies additional mental health service (20-50min)
Videoconference	93351	Focussed psychological strategies additional mental health service (20-50min)
Phone	93352	Focussed psychological strategies additional mental health service (20-50min)
In-person	93353	Focussed psychological strategies additional mental health service (50min)
Videoconference	93354	Focussed psychological strategies additional mental health service (50min)
Phone	93355	Focussed psychological strategies additional mental health service (50min)
In-person	93375	Psychological therapy health service RACF assessment (30-50min)
In-person	93376	Psychological therapy health service RACF assessment (50min)
In-person	93381	Psychological therapy health service RACF assessment (20-50min)
In-person	93382	Psychological therapy health service RACF assessment (50min)

In-person	93512	Psychology health service RACF chronic disease (30min)
In-person	93535	Psychology health service RACF CDM (20min)
In-person	93557	Psychology health service RACF Aboriginal or Torres Strait Islander (30min)
In-person	93590	Psychology health service RACF Aboriginal or Torres Strait Islander assessment (20min)

Supplementary Table S13: Speech pathology

**Mode**	**Medicare Item Number**	**Title**
In-person	10970	Speech pathology CDM
In-person	82005	Speech pathology to under 25 yrs old with complex neurodevelopmental disorder or eligible disability (≥50min)
In-person	82020	Speech pathology to under 25 yrs old with complex neurodevelopmental disorder or eligible disability (≥30min) + report
In-person	81360	Speech pathology to Aboriginal or Torres Strait Islander (≥20min)
In-person	93513	Speech pathology RACF assessment
In-person	93536	Speech pathology RACF service
In-person	93558	Speech pathology Aboriginal or Torres Strait Islander RACF assessment
In-person	93591	Speech pathology Aboriginal or Torres Strait Islander RACF service

Supplementary Table S14: Other items extracted

**Mode**	**Medicare Item Number**	**Title**
In-person	80150	Focussed psychological strategies (social worker) in consultation room (20-50min)
Videoconference	80151	Focussed psychological strategies (social worker) (20-50min)
In-person	80154	Focussed psychological strategies (social worker) in consultation room to a person other than the patient (20-50min)
In-person	80155	Focussed psychological strategies (social worker) outside consultation room (20-50min)
In-person	80156	Focussed psychological strategies (social worker) outside consultation room to a person other than the patient (20-50min)
In-person	80160	Focussed psychological strategies (social worker) in consultation room (≥50min)
Videoconference	80161	Focussed psychological strategies (social worker) (50min)
In-person	80162	Focussed psychological strategies (social worker) in consultation room to a person other than the patient (≥50min)

In-person	80165	Focussed psychological strategies (social worker) outside consultation room (50min)
In-person	80166	Focussed psychological strategies (social worker) outside consultation room to a person other than the patient (≥50min)
In-person	80170	Focussed psychological strategies (social worker) group (≥60min) (4-10 patients)
Videoconference	80171	Focussed psychological strategies (social worker) group (≥60min) (4-10 patients)
In-person	80172	Focussed psychological strategies (social worker) group (≥90min) (4-10 patients)
Videoconference	80173	Focussed psychological strategies (social worker) group (≥90min) (4-10 patients)
In-person	80174	Focussed psychological strategies (social worker) group (≥120min) (4-10 patients)
Videoconference	80175	Focussed psychological strategies (social worker) group (≥120min) (4-10 patients)
In-person	81005	Pregnancy support counselling (social worker) (30min)
In-person	82030	Audiology, optometry, orthoptic or physiotherapy health service (≥50min) provided to a patient aged under 25 years with complex neurodevelopmental disorder or eligible disability
In-person	82035	Audiology, optometry, orthoptic or physiotherapy health service (≥30min) provided to a patient aged under 25 years with complex neurodevelopmental disorder or eligible disability + report
In-person	82376	Eating disorder psychological treatment service (20-50min) in consultation room (social worker)
Videoconference	82377	Eating disorder psychological treatment service (20-50min) (social worker)
In-person	82378	Eating disorder psychological treatment service (20-50min) outside consultation room (social worker)
In-person	82379	Eating disorder psychological treatment service (≥50min) in consultation room (social worker)
Videoconference	82380	Eating disorder psychological treatment service (≥50min) (social worker)
In-person	82381	Eating disorder psychological treatment service (≥50min) outside consultation room (social worker)
In-person	82382	Eating disorder psychological treatment service (≥60min) group
Videoconference	82383	Eating disorder psychological treatment service (≥60min) group
In-person	91150	Focussed psychological strategies (social worker) in consultation rooms (20-50min) - bushfire
Videoconference	91151	Focussed psychological strategies (social worker) (20-50min) - bushfire
In-person	91155	Focussed psychological strategies (social worker) outside consultation rooms (20-50min) - bushfire
In-person	91160	Focussed psychological strategies (social worker) in consultation rooms (50min) - bushfire
Videoconference	91161	Focussed psychological strategies (social worker) (50min) - bushfire
In-person	91165	Focussed psychological strategies (social worker) outside consultation rooms (50min) - bushfire
Videoconference	91175	Focussed psychological strategies (social worker) (20-50min)
Videoconference	91176	Focussed psychological strategies (social worker) (≥50min)
Videoconference	91196	Focussed psychological strategies (social worker) to someone other than the patient (20-50min)
Videoconference	91197	Focussed psychological strategies (social worker) to someone other than the patient (≥50min)

Phone	91187	Focussed psychological strategies (social worker) + report (20-50min)
Phone	91188	Focussed psychological strategies (social worker) + report (≥50min)
Phone	91204	Focussed psychological strategies (social worker) to someone other than the patient (20-50min)
Phone	91205	Focussed psychological strategies (social worker) to someone other than the patient (≥50min)
Videoconference	93026	Pregnancy support counselling health service provided to a person who is currently pregnant or who has been pregnant in the preceding 12 months by an eligible psychologist, eligible social worker or eligible mental health nurse (≥30min)
Phone	93029	Pregnancy support counselling health service provided to a person who is currently pregnant or who has been pregnant in the preceding 12 months by an eligible psychologist, eligible social worker or eligible mental health nurse (≥30min)
Videoconference	93033	Speech pathology, occupational therapy, audiology, optometry, orthoptic or physiotherapy health service provided to a patient aged under 25 years with complex neurodevelopmental disorder or eligible disability (≥50min)
Videoconference	93036	Speech pathology, occupational therapy, audiology, optometry, orthoptic or physiotherapy health service provided by telehealth attendance to a patient aged under 25 years for the treatment of a diagnosed complex neurodevelopmental disorder (≥30min) + report
Phone	93041	Speech pathology, occupational therapy, audiology, optometry, orthoptic or physiotherapy health service provided to a patient aged under 25 years with complex neurodevelopmental disorder or eligible disability (≥50min)
Phone	93044	Speech pathology, occupational therapy, audiology, optometry, orthoptic or physiotherapy health service provided by telehealth attendance to a patient aged under 25 years for the treatment of a diagnosed complex neurodevelopmental disorder (≥30min) + report
Videoconference	93100	Eating disorder psychological treatment (20-50min) by social worker
Videoconference	93103	Eating disorder psychological treatment (≥50min) by social worker
Phone	93134	Eating disorder psychological treatment (20-50min) by social worker
Phone	93137	Eating disorder psychological treatment (≥50min) by social worker
In-person	93326	Focussed psychological strategies RACF (social worker) (20-50min)
In-person	93327	Focussed psychological strategies RACF (social worker) (50min)
In-person	93362	Focussed psychological strategies (social worker) additional mental health in consultation room (20-50min)
Videoconference	93363	Focussed psychological strategies (social worker) additional mental health (20-50min)
Phone	93364	Focussed psychological strategies (social worker) additional mental health (20-50min)
In-person	93365	Focussed psychological strategies (social worker) additional mental health in consultation room (50min)
Videoconference	93366	Focussed psychological strategies (social worker) additional mental health (50min)

Phone	93367	Focussed psychological strategies (social worker) additional mental health (50min)
In-person	93385	Focussed psychological strategies (social worker) RACF mental health plan (20-50min)
In-person	93386	Focussed psychological strategies (social worker) RACF mental health plan (50min)
In-person	81005	Pregnancy support counselling health service provided to a patient, who is currently pregnant or who has been pregnant in the preceding 12 months, by an eligible social worker (≥30min)
Videoconference	93592	Social worker RACF Aboriginal or Torres Strait Islander service
Phone	93593	Social worker RACF Aboriginal or Torres Strait Islander service

Supplementary Table S15: Combined allied health telehealth item numbers

Mode	Medicare Item Number	Title
Videoconference	93000	AH CDM telehealth COVID
Videoconference	93048	AH Aboriginal or Torres Strait Islander VC
Videoconference	93537	AH RACF VC
Videoconference	93592	AH RACF Aboriginal or Torres Strait Islander VC
Phone	93013	AH CDM phone COVID
Phone	93061	AH Aboriginal or Torres Strait Islander phone
Phone	93538	AH RACF phone
Phone	93593	AH RACF Aboriginal or Torres Strait Islander phone

Supplementary Material 2 Quarterly Claimed Services through the Medicare Benefits Schedule from Q1 2017 to Q4 2024, Segmented into In-person, Videoconferencing, Telephone and Telehealth (Videoconferencing + Telephone) Modalities for All Allied Health Practitioners

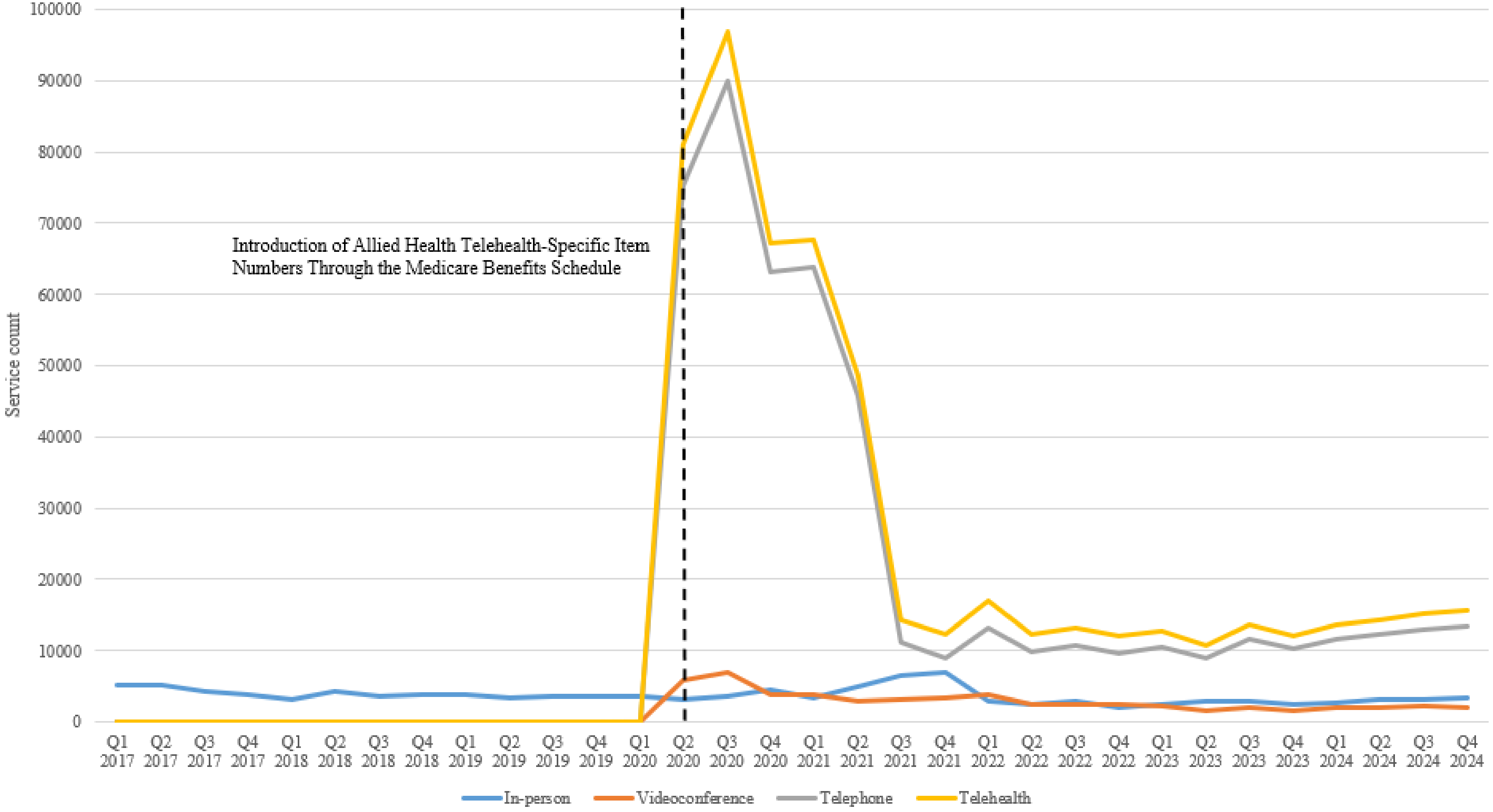

Supplementary Figure S1 Aboriginal or Torres Strait Islander Health Service

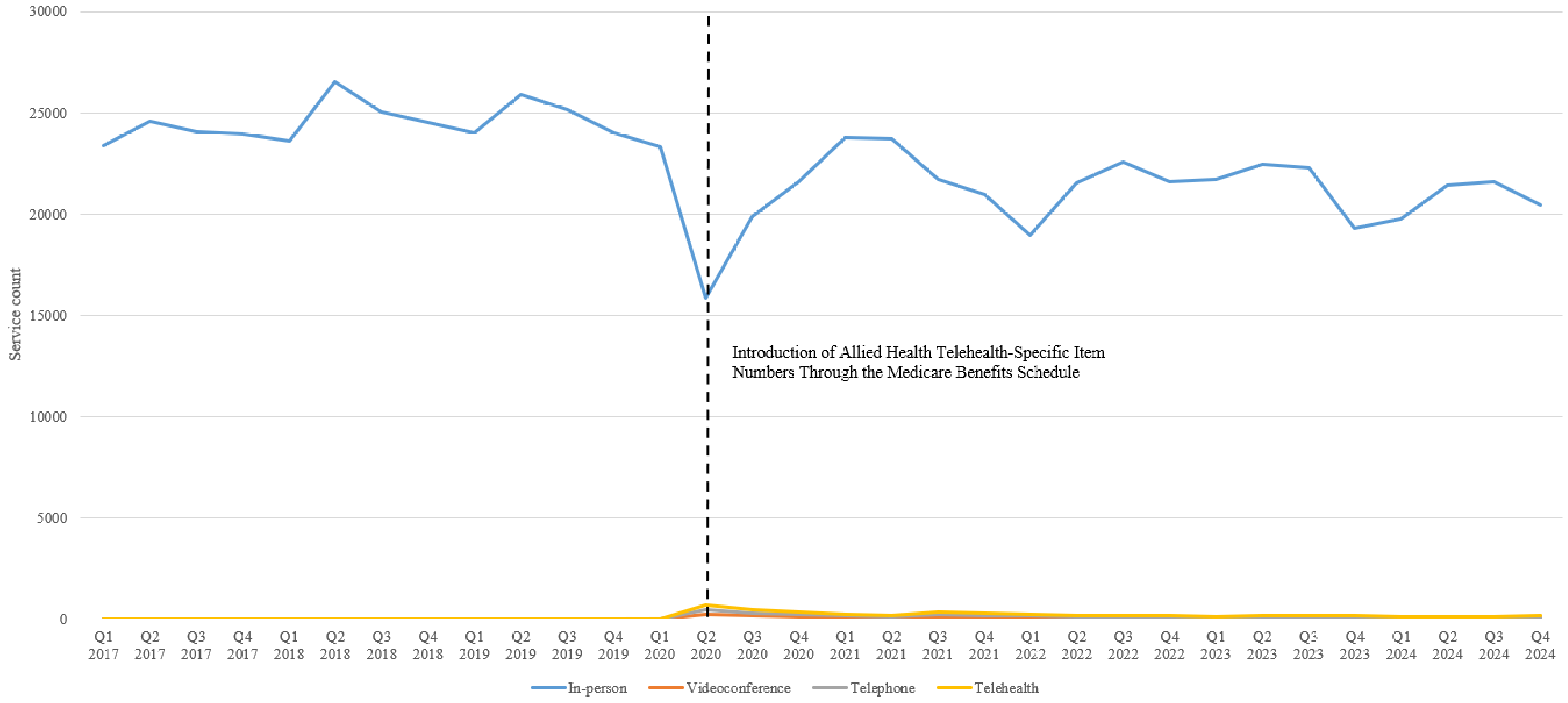

Supplementary Figure S2 Diabetes Education

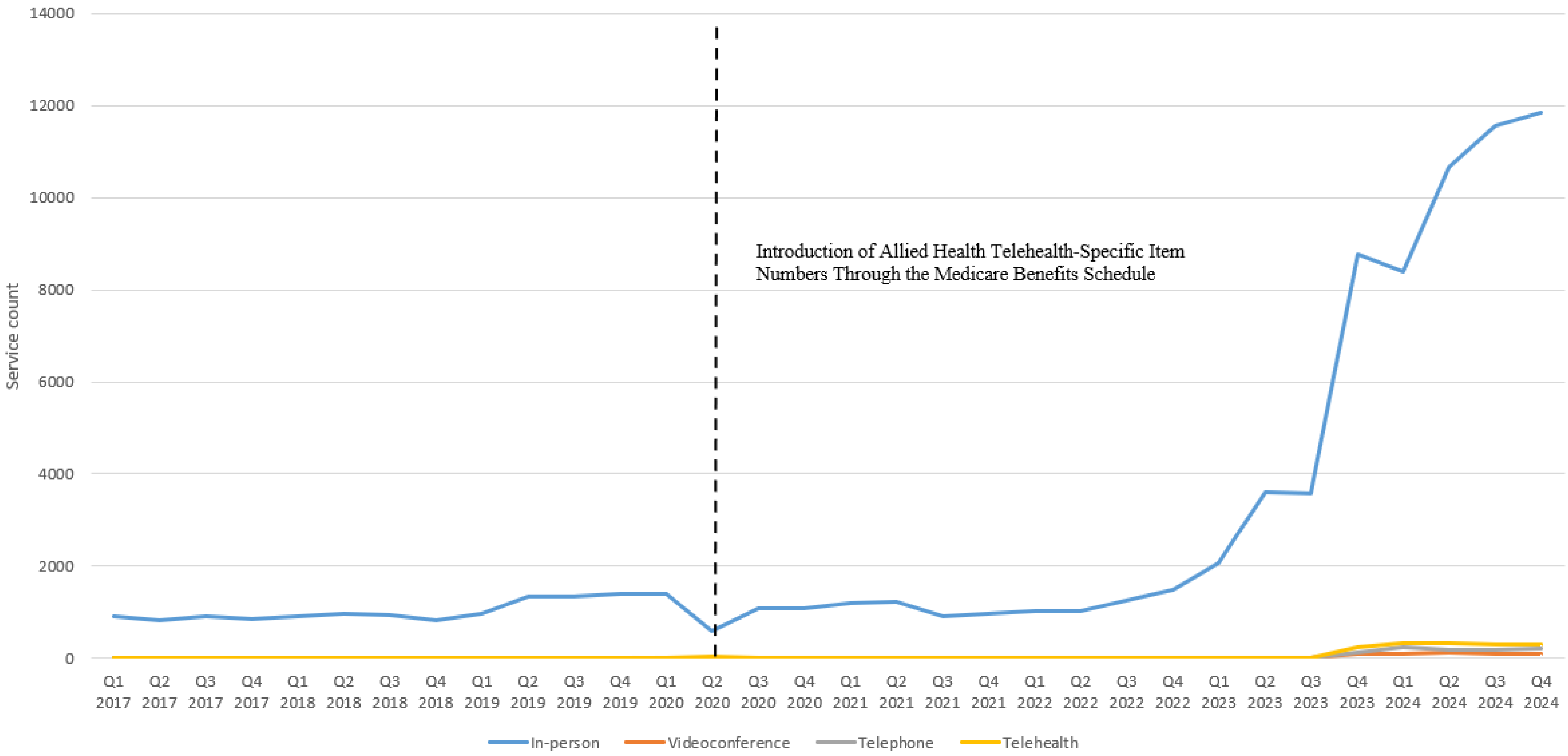

Supplementary Figure S3 Audiology

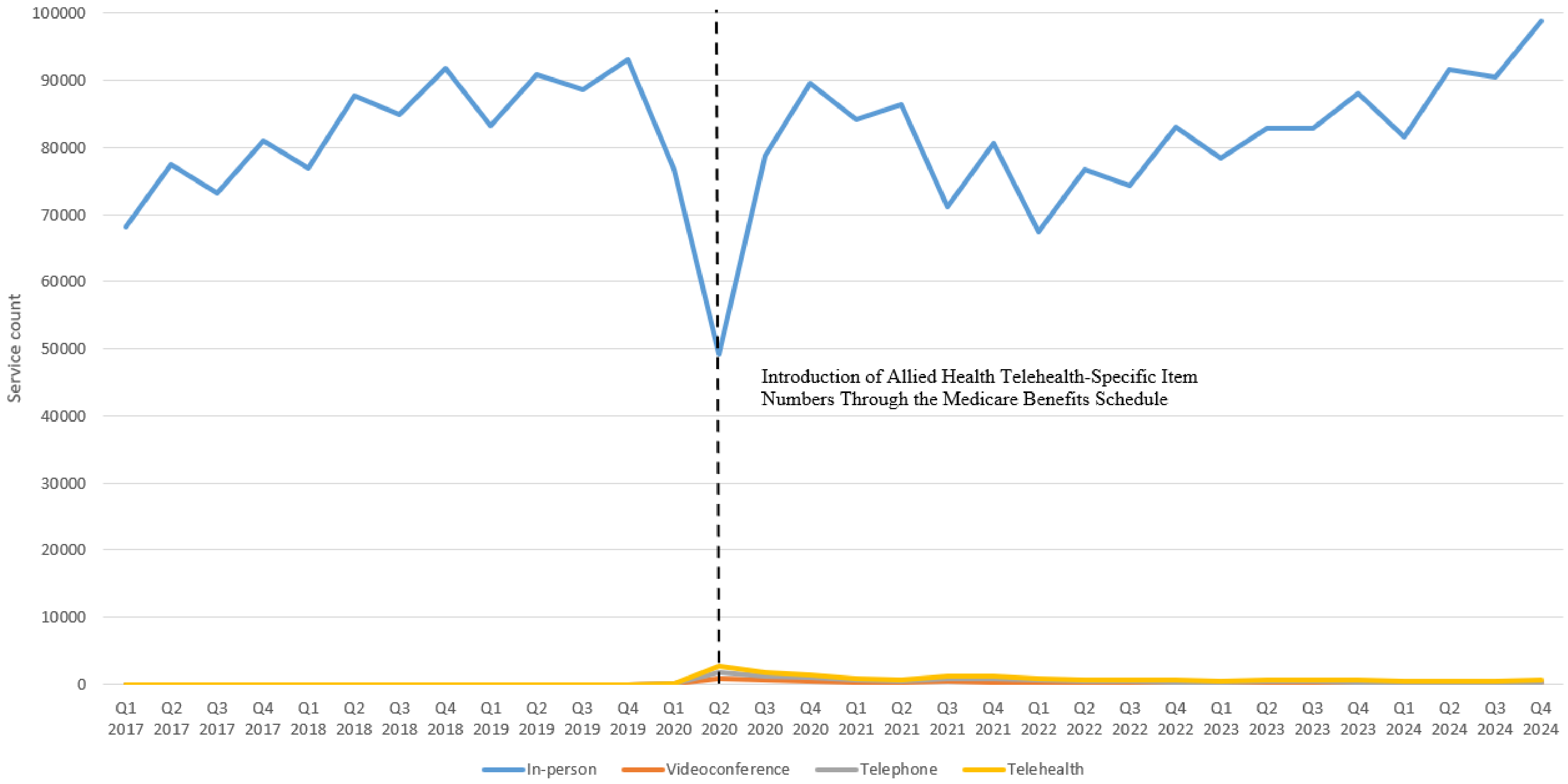

Supplementary Figure S4 Exercise Physiology

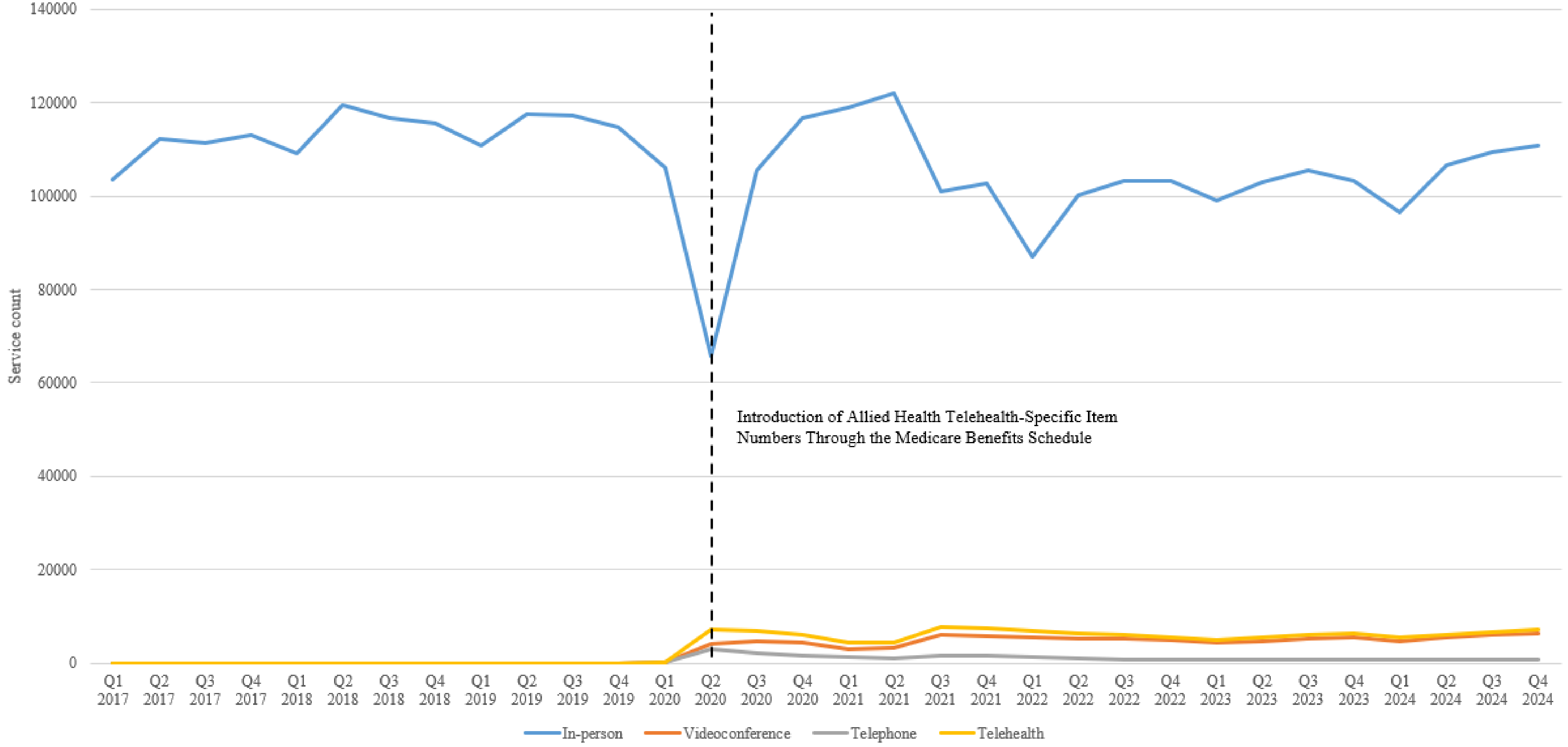

Supplementary Figure S5 Dietetics

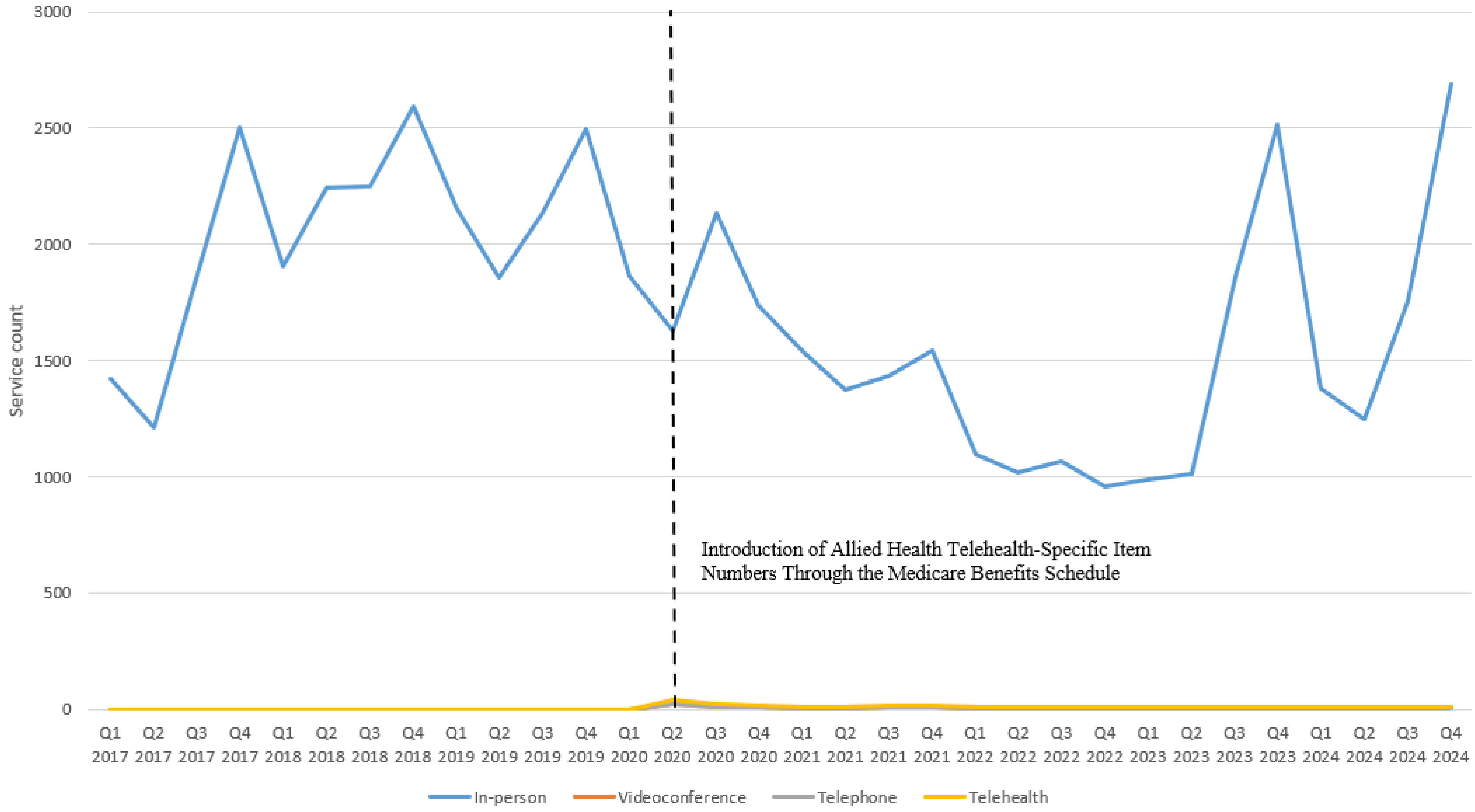

Supplementary Figure S6 Mental Health Services

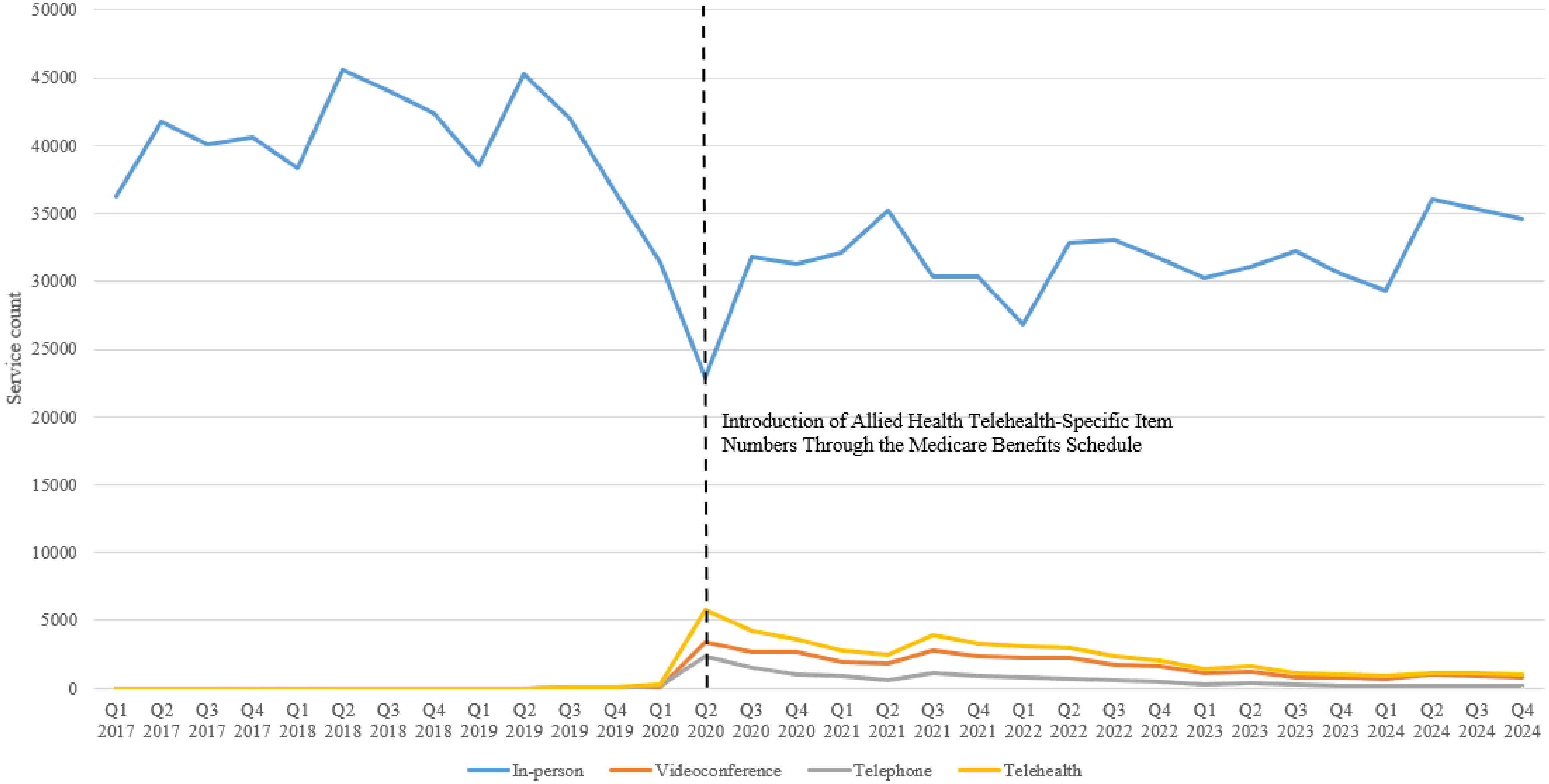

Supplementary Figure S7 Occupational Therapy

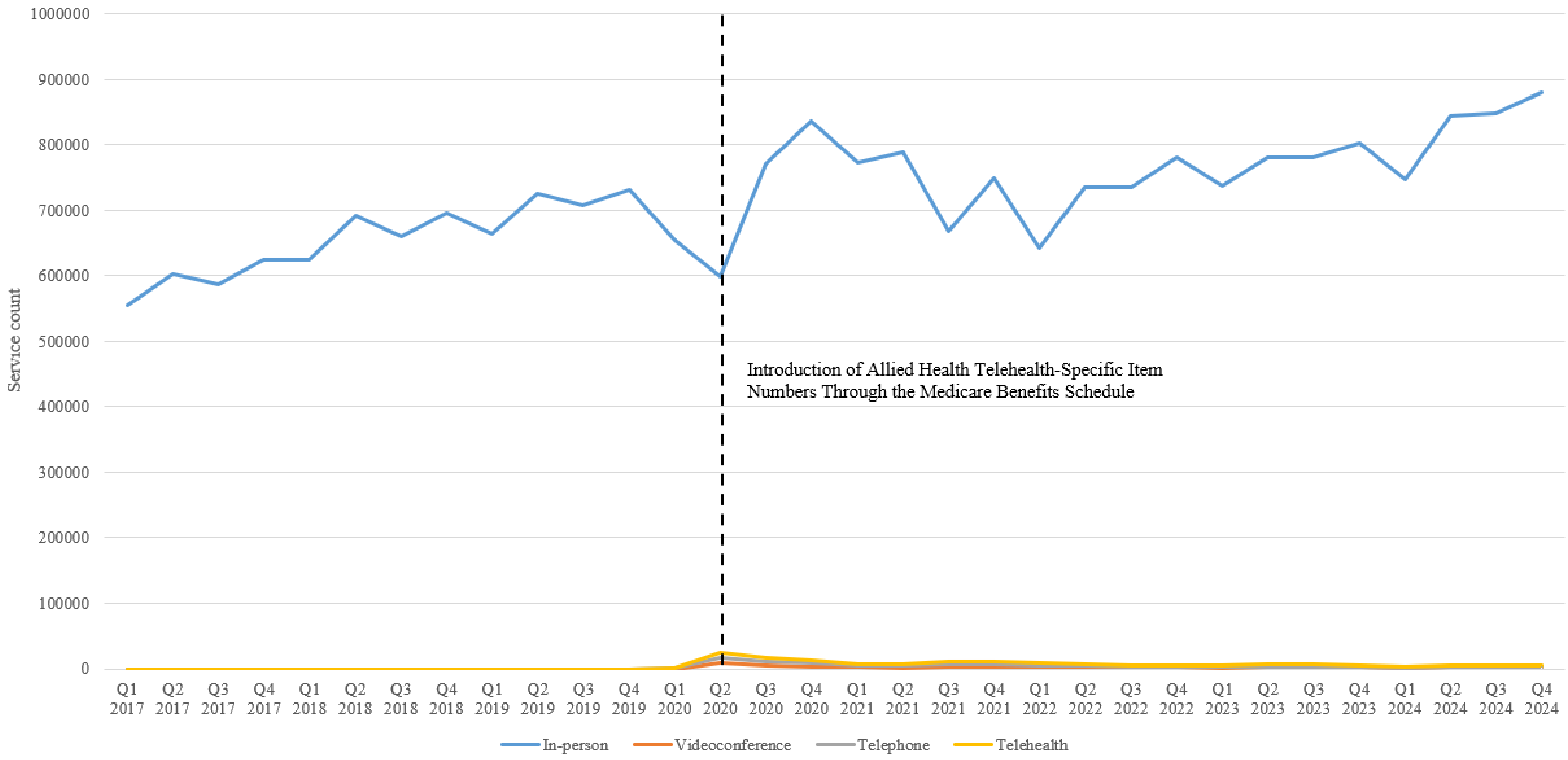

Supplementary Figure S8 Physiotherapy

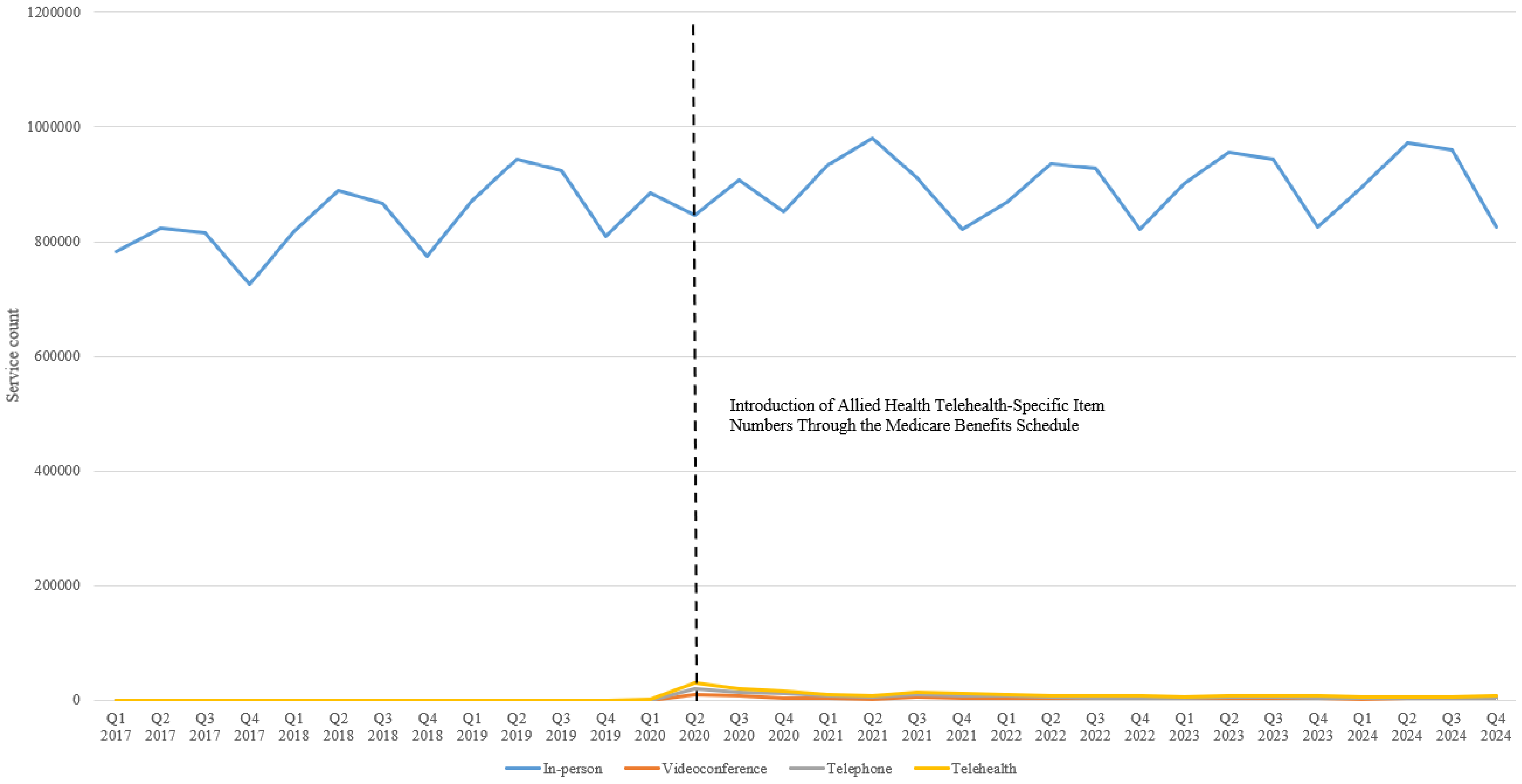

Supplementary Figure S9 Podiatry

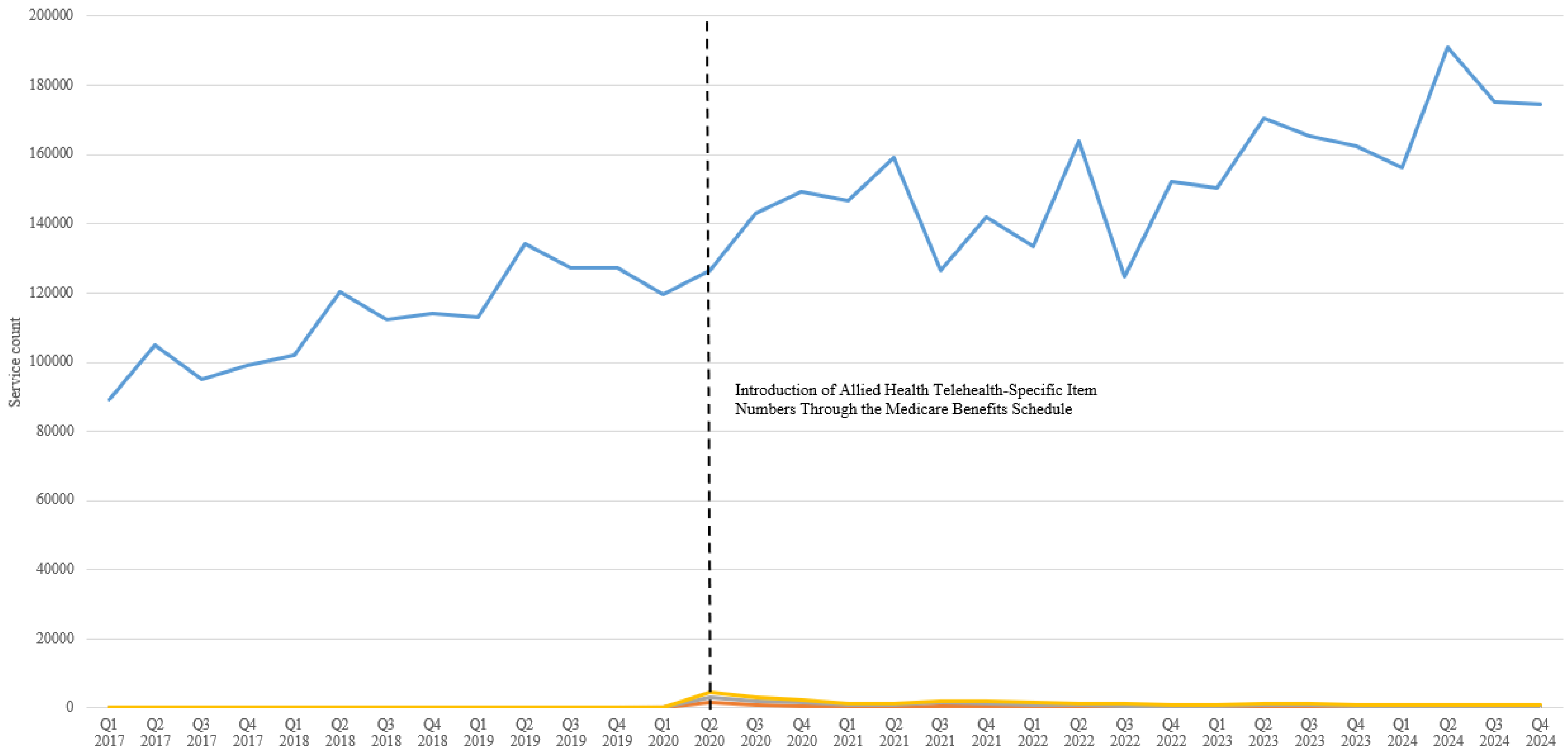

Supplementary Figure S10 Chiropractic

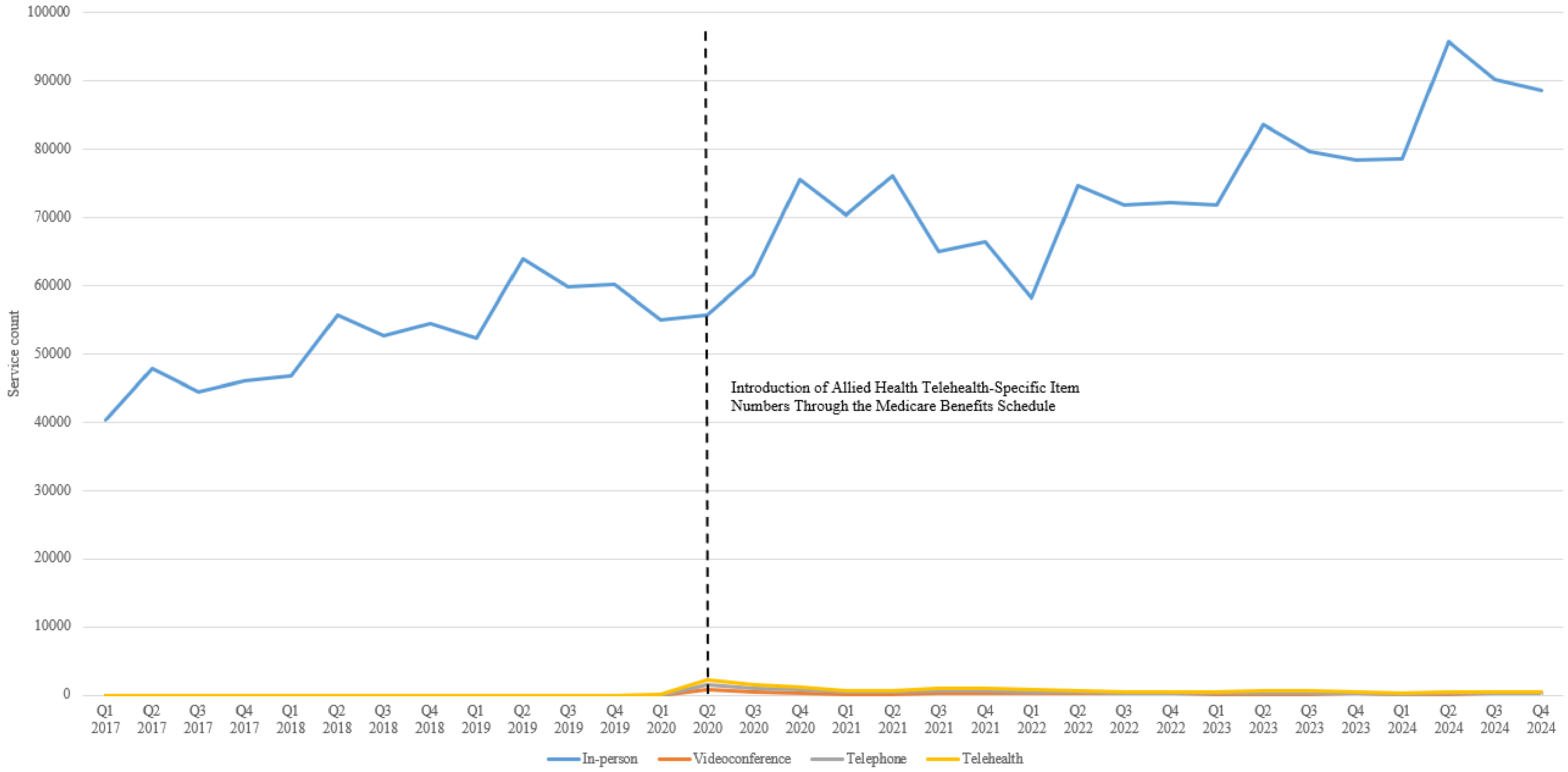

Supplementary Figure S11 Osteopathy

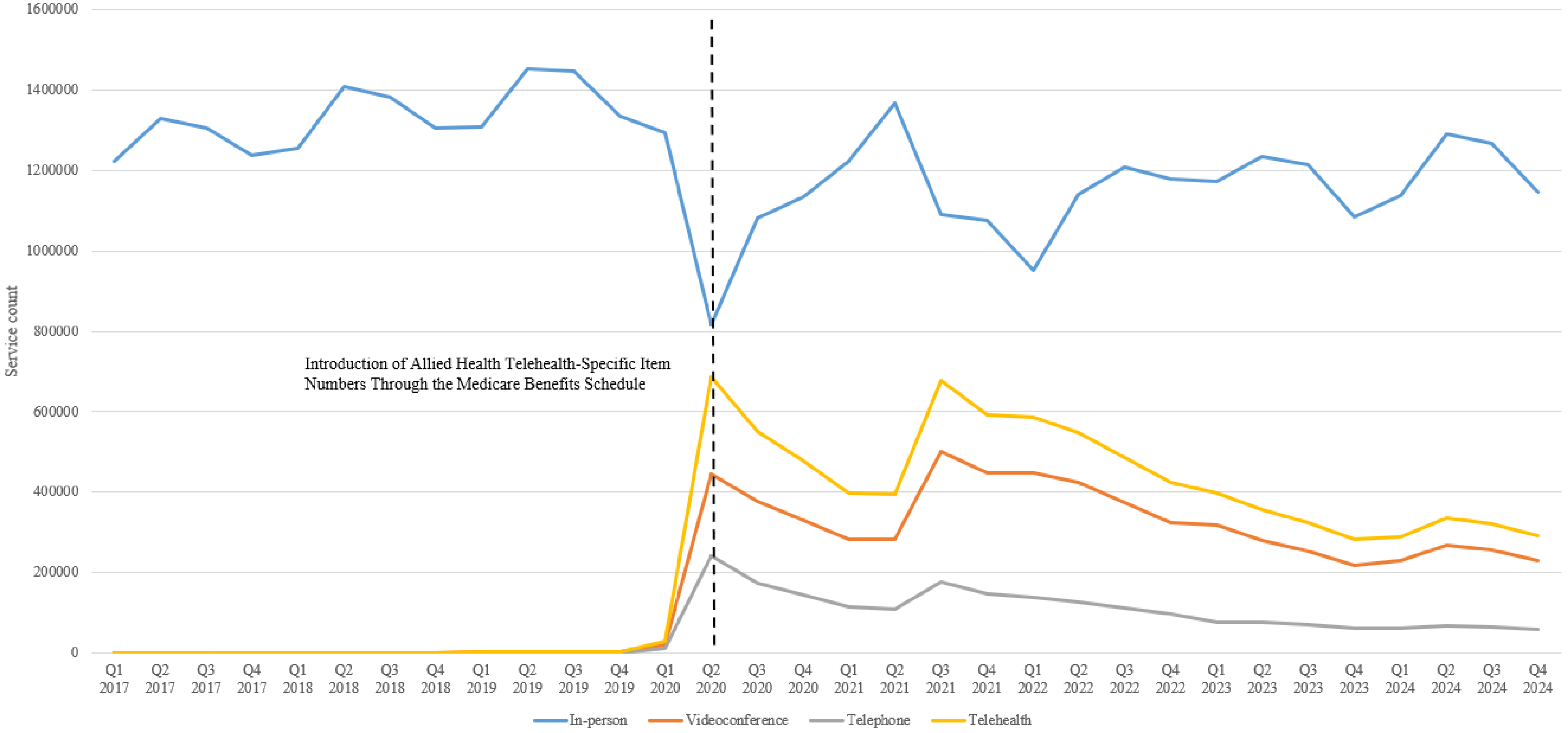

Supplementary Figure S12 Psychology

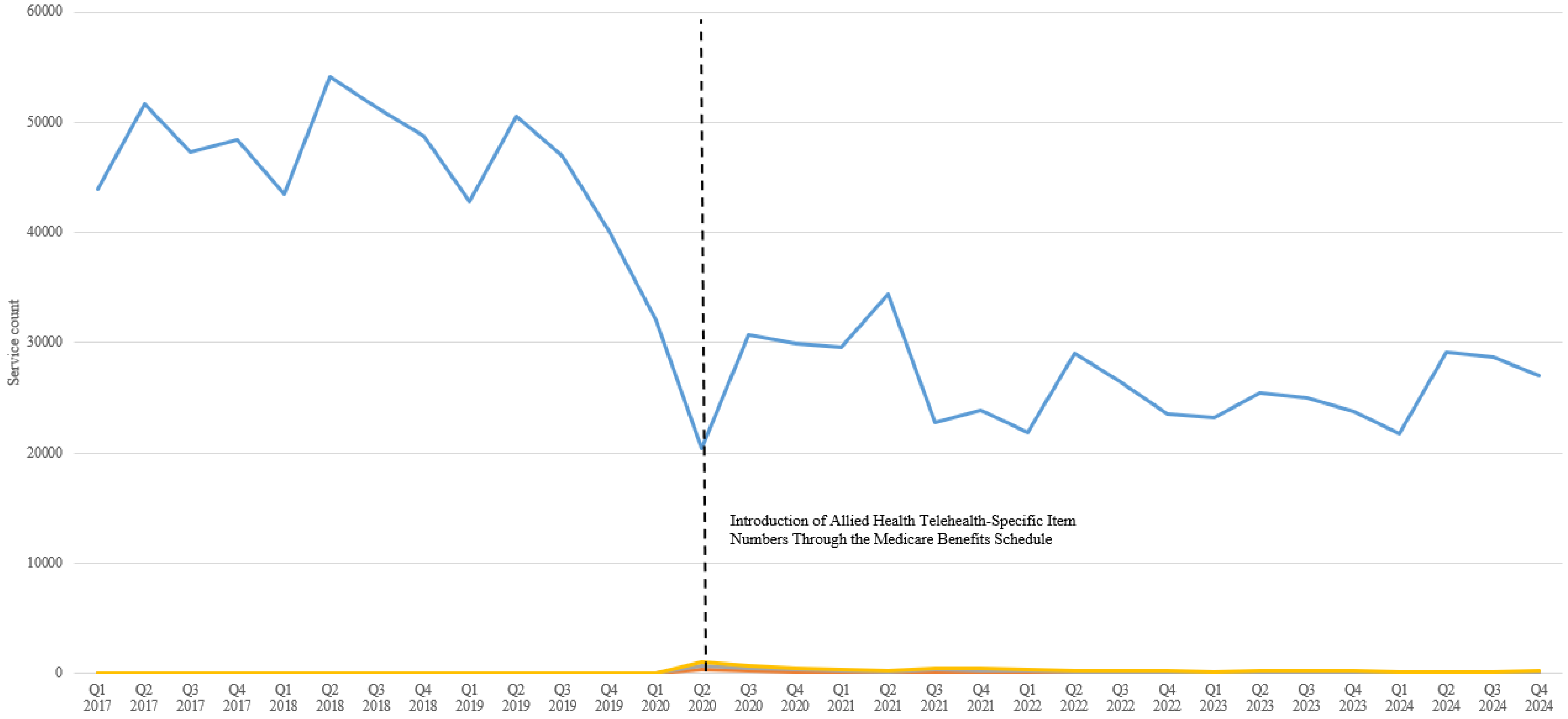

Supplementary Figure S13 Speech Pathology

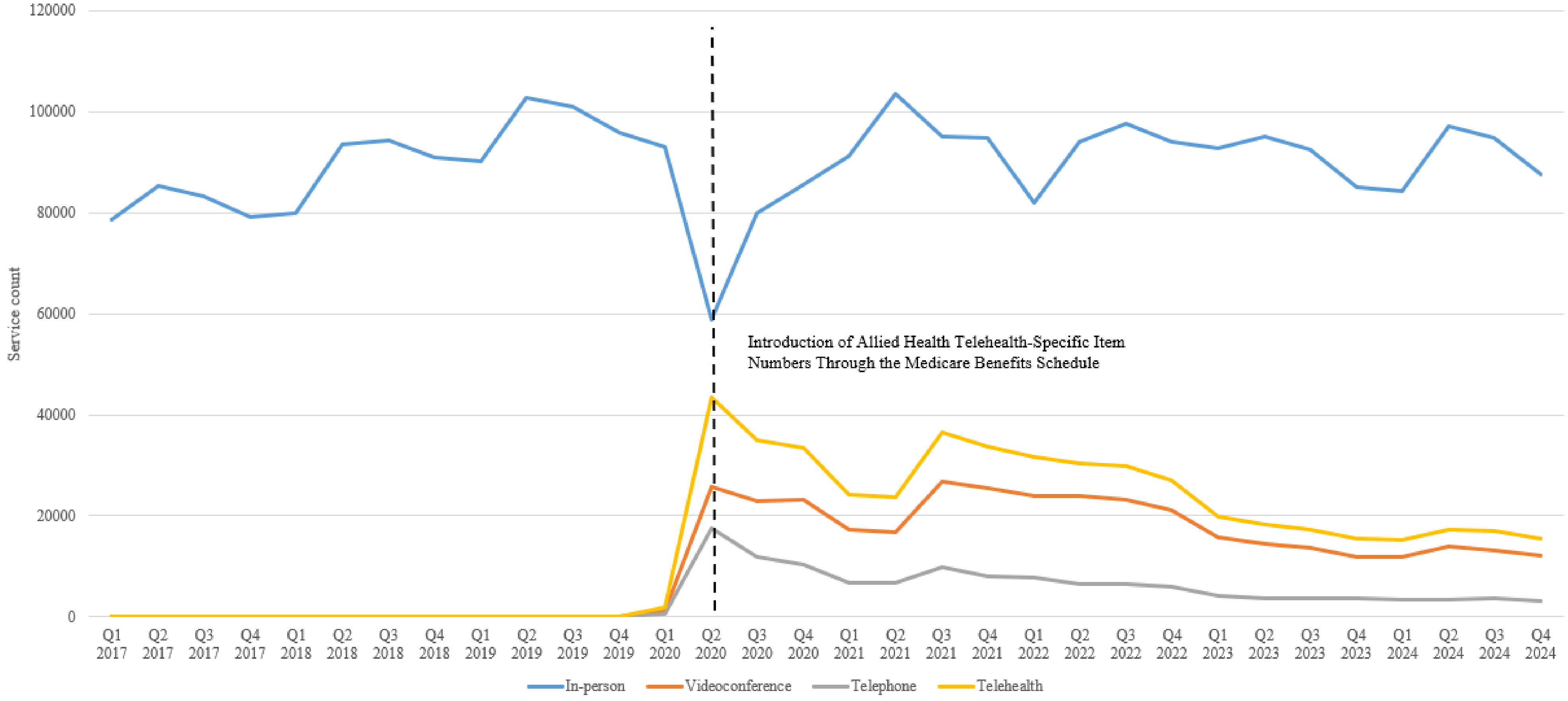

Supplementary Figure S14 Social Work
